# Pro-arrhythmogenic effects of atrial fibrillation-induced electrical remodelling: insights from the three-dimensional virtual human atria

**DOI:** 10.1113/jphysiol.2013.254987

**Published:** 2013-06-03

**Authors:** Michael A Colman, Oleg V Aslanidi, Sanjay Kharche, Mark R Boyett, Clifford Garratt, Jules C Hancox, Henggui Zhang

**Affiliations:** 1 Biological Physics Group, School of Physics and Astronomy Manchester M13 9PL, UK; 2 Cardiovascular Medicine Group, School of Medicine, University of Manchester Manchester M13 9PL, UK; 3 Department of Biomedical Engineering, Division of Imaging Sciences and Biomedical Engineering, King's College London London SE1 7EH, UK; 4 Manchester Heart Centre, Manchester Royal Infirmary Manchester M13 9PL, UK; 5 Department of Physiology and Pharmacology and Cardiovascular Research Laboratories, School of Medical Sciences, University Walk Bristol BS8 1TD, UK; 6 School of Computer Science and Technology, Harbin Institute of Technology Harbin, China

## Abstract

Chronic atrial fibrillation (AF) is associated with structural and electrical remodelling in the atria, which are associated with a high recurrence of AF. Through biophysically detailed computer modelling, this study investigated mechanisms by which AF-induced electrical remodelling promotes and perpetuates AF. A family of Courtemanche–Ramirez–Nattel variant models of human atrial cell action potentials (APs), taking into account of intrinsic atrial electrophysiological properties, was modified to incorporate various experimental data sets on AF-induced changes of major ionic channel currents (*I*_CaL_, *I*_Kur_, *I*_to_, *I*_K1_, *I*_Ks_, *I*_NaCa_) and on intracellular Ca^2+^ handling. The single cell models for control and AF-remodelled conditions were incorporated into multicellular three-dimensional (3D) atrial tissue models. Effects of the AF-induced electrical remodelling were quantified as the changes of AP profile, AP duration (APD) and its dispersion across the atria, and the vulnerability of atrial tissue to the initiation of re-entry. The dynamic behaviour of re-entrant excitation waves in the 3D models was characterised. In our simulations, AF-induced electrical remodelling abbreviated atrial APD non-uniformly across the atria; this resulted in relatively short APDs co-existing with marked regional differences in the APD at junctions of the crista terminalis/pectinate muscle, pulmonary veins/left atrium. As a result, the measured tissue vulnerability to re-entry initiation at these tissue junctions was increased. The AF-induced electrical remodelling also stabilized and accelerated re-entrant excitation waves, leading to rapid and sustained re-entry. Under the AF-remodelled condition, re-entrant scroll waves in the 3D model degenerated into persistent and erratic wavelets, leading to fibrillation. In conclusion, realistic 3D atrial tissue models indicate that AF-induced electrical remodelling produces regionally heterogeneous and shortened APD; these respectively facilitate initiation and maintenance of re-entrant excitation waves.

**Key points**

- Previous studies have shown that atrial electrical properties are altered (remodelled) by atrial fibrillation (AF) and that the recurrence of AF is high following remodelling. However, demonstrating a causal link between atrial remodelling in experimental models and the increased risk of AF is a challenge.
- AF-induced electrical remodelling abbreviated atrial action potential duration (APD) non-uniformly across the atria; this resulted in relatively short APDs co-existing with marked regional differences in the APD at junctions of the crista terminalis/pectinate muscle, pulmonary veins/left atrium.
- It increases tissue vulnerability to re-entry initiation and maintenance at these tissue junctions.
- The AF-induced electrical remodelling also stabilized and accelerated re-entrant excitation waves, leading to rapid and sustained re-entry.
- This study provides novel insights towards understanding the mechanisms underlying the pro-arrhythmic effects of the AF-induced electrical remodelling in atrial tissue.

## Introduction

Atrial fibrillation (AF) is the most common cardiac arrhythmia (Nattel *et al.* 2005), affecting approximately 6 million adults in Europe (Camm *et al.* 2010). Rates of hospitalisation due to AF are increasing in epidemic proportions (Stewart *et al.* 2004; Anter *et al.* 2009). AF contributes significantly to morbidity, and can predispose to stroke, heart failure and even sudden death (Benjamin *et al.* 1998; Anter *et al.* 2009). The disorder is characterised by rapid and irregular electrical activation of the atria that over-rides normal sinus rhythm (SR) and leads to reduced cardiac output. AF is thought to be sustained by the presence of either rapid focal activity or driving re-entrant rotor waves (Jalife *et al.* 2002; Rosanio *et al.* 2004; Nattel *et al.* 2005). Electrical activity during AF has been clinically (Sanders *et al.* 2005) and experimentally (Mansour *et al.* 2001) characterised by a dominant frequency in the left atrium (LA) and multiple frequencies in the right atrium (RA). These observations support a hypothesis that AF can be driven by a single rotor wave in the LA and multiple re-entrant wavelets in the RA (Haïssaguerre *et al.* 1998; Chen *et al.* 1999).

The atria are characterised by a large degree of regional differences in the cellular electrical properties (Feng *et al.* 1998; Li *et al.* 2001; Burashnikov *et al.* 2004; Gong *et al.* 2008), which may be pro-arrhythmic. Regions of the pulmonary veins (PVs) with the left atrial wall in the LA, and the crista terminalis (CT) with the pectinate muscles (PM) in the RA, characterised by large regional differences in action potential (AP) properties and structural anisotropy, have been identified as the high-frequency excitation sources underlying AF (Cox *et al.* 1991; Haïssaguerre *et al.* 1998; Mandapati *et al.* 2000; Arora *et al.* 2003; Kumagai *et al.* 2004; Sanders *et al.* 2005). In AF patients, such sources are most commonly observed primarily in the PV/LA region. Recent modelling studies have demonstrated the role of atrial regional AP heterogeneity and anisotropy in the initiation of re-entrant waves (Aslanidi *et al.* 2009) and their subsequent breakdown into multiple re-entrant wavelets (Aslanidi *et al.* 2011*b*, 2013*a*; Butters *et al.* 2013).

The intrinsic tissue properties of the atria can be altered during AF due to AF-induced electrical and structural remodelling (e.g. Ausma *et al.* 1997*a*; van Wagoner *et al.* 1999; Bosch *et al.* 1999; Workman *et al.* 2001; van der Velden & Jongsma, 2002; Dobrev & Ravens, 2003; Severs *et al.* 2008). Multiple experimental studies have shown that AF-induced electrical remodelling is characterised by an abbreviated atrial AP morphology, which is associated with underlying changes to the density and kinetics of some membrane ion channels and to cellular Ca^2+^ handling processes (van Wagoner *et al.* 1997, 1999; Bosch *et al.* 1999; Workman *et al.* 2001; Dobrev & Ravens, 2003; Wettwer *et al.* 2004; Christ *et al.* 2008; Neef *et al.* 2010; Voigt *et al.* 2012; Fuente *et al.* 2013). Atrial structural remodelling has been characterised by down-regulation and heterogeneous expression of connexin proteins that form intercellular gap junctions (responsible for the AP conduction), as well as the presence of severe fibrosis, accumulation of fatty deposits and fibre disorganisation (Ausma *et al.* 1997*a*,*b*; van Wagoner *et al.* 1999; van Wagoner & Nerbonne, 2000; Polontchouk *et al.* 2001; van der Velden & Jongsma, 2002; Severs *et al.* 2004, 2008; Bikou *et al.* 2011). All of these factors may contribute to decreases in the AP conduction velocity and increases in conduction anisotropy and heterogeneity.

AF remodelling may be responsible for an increased susceptibility to atrial arrhythmia (Fareh *et al.* 1998). Catheter ablation treatment can effectively terminate AF in the short term (Calkins *et al.* 2012), but high AF recurrence rates are seen in patients with chronic AF (Weerasooriya *et al.* 2011) associated with high levels of AF remodelling. In a homogeneous two-dimensional (2D) atrial model (Pandit *et al.* 2005), the pro-arrhythmic role of reduced atrial AP duration (APD) and conduction velocity has been demonstrated, as both contribute to a decrease in atrial excitation wavelength, which is the product of the conduction velocity and the effective refractory period (ERP). The latter effectively increases the atrial substrate size necessary for the initiation and maintenance of re-entrant circuits (Pandit *et al.* 2005). However, the functional effect of AF-induced electrical remodelling on the regional dispersion of atrial electrical properties – which can play an important role in arrhythmogenesis (Fareh *et al.* 1998; Aslanidi *et al.* 2011*b*, 2013*a*) – has not yet been fully characterised. Characterising this relationship, through the use of computational modelling, has the potential to help understand better the mechanisms underlying increased AF risks in AF-remodelled atria.

In this study, we have applied the previously developed 3D virtual human atria (Aslanidi *et al.* 2011*b*; Kharche *et al.* 2012) to characterise the functional role of AF-induced electrical remodelling on atrial electrical properties and excitation dynamics. First, the Courtemanche–Ramirez–Nattel (CRN) model (Courtemanche *et al.* 1998) for the human atrial AP has been updated to incorporate recently developed formulations of outward K^+^ currents (Maleckar *et al.* 2009) and of intracellular Ca^2+^ dynamics (Koivumäki *et al.* 2011). This updated model has then been used to construct a family of electrophysiologically detailed regional atrial cell models, accounting for the intrinsic cellular electrical heterogeneities within the atria. The single cell models have then been modified to included four distinctive models of AF-induced electrical remodelling, which incorporate experimental data from a range of datasets.

The single cell models are incorporated into a 3D anatomical model of the human atria developed in a previous study (Aslanidi *et al.* 2011*b*; Kharche *et al.* 2012), which is updated to include further segmentations of distinctive regions, in particular the PVs from the LA. Using the 3D model, we have explored the effects of AF-induced electrical remodelling on: (1) the intrinsic APD heterogeneity in single cells and across the entire intact 3D atria; (2) the vulnerability of tissue to unidirectional conduction block and the initiation of re-entry in response to an S1–S2 pacing protocol at the CT/PM and LA/PV junctions; and (3) the long-term development and breakdown of re-entrant excitation waves during AF. We also identify mechanisms by which a series of localised rapid stimuli (mimicking rapid atrial pacing, as used in previous experimental studies; Morillo *et al.* 1995; Ausma *et al.* 1997*b*; Sun *et al.* 1998; van Wagoner & Nerbonne, 2000) applied to the atrial wall may lead to the development of re-entrant circuits and AF.

## Methods

### Single atrial cell models

The CRN model for the human atrial AP was chosen as its basic formulation incorporates human atrial cell data and reproduces human atrial AP morphology and rate dependence (Courtemanche *et al.* 1998). The model has been modified to consider regional AP heterogeneity within the atria (Seemann *et al.* 2006; Aslanidi *et al.* 2011*b*; Colman *et al.* 2011; Dorn *et al.* 2012), and has been used extensively to study re-entrant arrhythmias in the human atrium (e.g. Pandit *et al.* 2005; Seemann *et al.* 2006; Kharche & Zhang, 2008; Aslanidi *et al.* 2011*b*; Kharche *et al.* 2012). In this study, we further updated the CRN model by incorporating recent advances in atrial cell electrophysiology model development, such as new formulations for the transient outward current (*I*_to_) and the ultra-rapid potassium current (*I*_Kur_) (Maleckar *et al.* 2009) and for intracellular Ca^2+^ handling developed in the Koivumäki *et al.* (KM) model (Koivumäki *et al.* 2011). To improve computational efficiency, we used two compartments rather than four compartments for representing Ca^2+^ cycling in the sarcoplasmic reticulum in the KM model (Online Supplement Fig. S1). Such a simplified Ca^2+^ handling system increases computational efficiency by ∼50% while preserving the characteristics of the intracellular Ca^2+^ transient in the peripheral and interior sites of the cell as seen in the original KM model. As electrogenesis of the sodium–calcium exchanger current (*I*_NaCa_) and L-type calcium channel current (*I*_CaL_) in the KM model is dependent on the Ca^2+^ transient in the peripheral site of the cell, such a simplification has no significant effect on the genesis of the AP. Therefore, such a simplification is reasonable as the focus of the present study was to investigate the effects of the AF-induced electrical remodelling on arrhythmogenesis related to APD abbreviation and its regional heterogeneity. Whereas the effect of remodelling on the dynamics of intracellular Ca^2+^ cycling may also have pro-arrhythmic properties in its own right, it is not the focus of the present study. Details of the model development are presented in the Online Supplement.

### Regional differences in cellular AP models

The updated CRN model was used as a base model that describes the RA cell. The model was then further modified to take into consideration the difference in the intrinsic electrophysiological properties of various atrial cell types. These modifications are based on experimental data from human (where data are available) and large animals (where human data are not available), an approach that is consistent with methods used in previous modelling studies (Seemann *et al.* 2006; Dorn *et al.* 2012). Briefly, the current densities of *I*_CaL_, *I*_to_ and *I*_Kur_ of the base model were scaled based on the data of Feng *et al.* (1998) to generate the CT, PM and atrio-ventricular ring (AVR) cell models. For right atrial appendage (RAA) and atrial septum (AS) cell models, the current formulations of *I*_CaL_, *I*_to_, *I*_Kur_ and the inwardly rectifying potassium current (*I*_K1_) were modified based on the data of Gong *et al.* (2008). For the LA model, the current formulations of the rapid delayed rectifier current (*I*_Kr_), the slow delayed rectifier current (*I*_Ks_) and *I*_to_ were modified based on data from Li *et al.* (2001) and Ehrlich *et al.* (2003). For the left atrial appendage (LAA) model, the current formulations of *I*_Kr_, *I*_Ks_, *I*_to_ and *I*_Kur_ were modified based on data from Caballero *et al.* (2010). For PV cells, the Jones *et al.* model (derived previously from the CRN cell model; Jones *et al.* 2012) was implemented. The Bachmann's bundle (BB) model was derived from a previous canine model (Aslanidi *et al.* 2011*a*), involving modifications to *I*_CaL_, *I*_to_ and *I*_K1_. Further details of the regional cell models are provided in the Online Supplement, where a summary of changes to ionic currents for each model can be found in Table S2.

### Simulation of AF-induced electrical remodelling

Remodelling in the ionic channel properties (such as the current density and channel kinetics) by AF has been well characterised (van Wagoner *et al.* 1997, 1999; Bosch *et al.* 1999; Workman *et al.* 2001; Dobrev & Ravens, 2003; Wettwer *et al.* 2004; Voigt *et al.* 2012). All these studies have shown significant decreases of the APD values at 90% repolarisation (APD_90_) due to remodelling, but the exact degree of the APD_90_ reduction varies considerably between studies, from 23 to 60% (Bosch *et al.* 1999; Dobrev & Ravens, 2003). The identified remodelling of ionic channels are also different among these studies: whereas remodelling in *I*_CaL_, *I*_K1_ and *I*_to_ are identified in all studies, remodelling in other channels, such as *I*_Kur_, is absent in some studies (Bosch *et al.* 1999; Grammer *et al.* 2000; Workman *et al.* 2001), but present in others (van Wagoner *et al.* 1997; Brandt *et al.* 2000; Christ *et al.* 2004; Caballero *et al.* 2010; see Table 1 for details). Recent studies have also characterised changes to the subcellular Ca^2+^ handling properties of atrial myocytes associated with AF in human (Neef *et al.* 2010; Shanmugam *et al.* 2011; Voigt *et al.* 2012).

**Table 1:** Review of AF-induced remodelling data and model parameters

Process	Experimental observation	AF-1	AF-2	AF-3	AF-4
*I*_CaL_	−70%+ kinetic change (Bosch *et al.* 1999) −63% (van Wagoner *et al.* 1999) −72% (Skasa *et al.* 2001) −65% (Workman *et al.* 2001) −50%, +4 mV shift (Christ *et al.* 2004) −33% mRNA expression (Oh *et al.* 2010)	−70%τ_ac,inac_+60%	−65%	−63%	−70%
*I*_Kur_	−49% (van Wagoner *et al.* 1997) −55% (Brandt *et al.* 2000) −55% RA (Caballero *et al.* 2010) −45% LA (Caballero *et al.* 2010) −50% (Christ *et al.* 2004) No change (Bosch *et al.* 1999) No change (Grammer *et al.* 2000) No change (Workman *et al.* 2001)	—	—	−49%	−50%
*I*_to_	−66% (van Wagoner *et al.* 1997) −70%+ 16 mV shift (Bosch *et al.* 1999) −44% (Brandt *et al.* 2000) −65% (Workman *et al.* 2001) −50% RA (Caballero *et al.* 2010) −75% LA (Caballero *et al.* 2010) −80% (Grammer *et al.* 2000)	−70%+16 mV shift	−65%	−66%	−65%
*I*_K1_	+106% (van Wagoner *et al.* 1997) +100% (Bosch *et al.* 1999) +137% (Dobrev *et al.* 2001) +73% (Dobrev *et al.* 2002) +75% (Workman *et al.* 2001)	+100%	+75%	+106%	+100%
*I*_Ks_	+100% LA (Caballero *et al.* 2010) > + 100% RA (Caballero *et al.* 2010) −30% mRNA (Lai *et al.* 1999) +56% mRNA (Brundel *et al.* 2001*b*)	—	—	—	+100%
*I*_NaCa_	+43%± 14% (Neef *et al.* 2010) +67% (Schotten *et al.* 2002) +201% protein (El-Armouche *et al.* 2006) Increase (Voigt *et al.* 2012)	—	—	—	+55%
*I*_Kr_	−27% mRNA (Lai *et al.* 1999) −34% mRNA (Brundel *et al.* 2001*b*) −30% mRNA expression (Oh *et al.* 2010) No change – mRNA (Brundel *et al.* 2001*a*)	—	—	—	No change
SERCA	Increased SR uptake (Shanmugam *et al.* 2011)	—	—	—	+50%
**RyR**	Open probability increased 4-fold (Voigt *et al.* 2012) Increased release (Hove-Madsen *et al.* 2004)	—	—	—	+300%
SR Ca^2+^ leak	Increased (Voigt *et al.* 2012)	—	—	—	+25%

To incorporate such variations in experimental data into the models, four different scenarios (AF-1 – AF-4) have been considered here for simulating AF-induced electrical remodelling. This has enabled us to: (1) consider a broad range of experimental data on identified ion channel remodelling; (2) investigate the effects of varying degrees of remodelling; and (3) draw general conclusions in a model-independent manner. Each of the four AF scenarios is described in detail below.

‘AF-1’ is based on the dataset of Bosch *et al.* (1999), and ‘AF-2’ on the dataset of Workman *et al.* (2001). Both of these models involve modifications to the same three ion currents: *I*_CaL_ and *I*_to_ were decreased, and *I*_K1_ was increased. AF-1 involves changes to both the current densities and the kinetics, whereas AF-2 involves modifying the current densities only.

‘AF-3’ is based on the datasets of van Wagoner *et al*. (1997, 1999), who identified AF-induced changes to outward K^+^ currents and inward Ca^2+^ currents. Thus, AF-3 simulations included modifications of *I*_CaL_, *I*_to_ and *I*_K1_ which are similar to AF-1 and AF-2, but also included a reduction in *I*_Kur_.

‘AF-4’ is based on an extensive review of the literature (Table 1), and combines modifications to all the currents from AF-3 with additional modifications to *I*_Ks_, *I*_NaCa_ and subcellular Ca^2+^ handling processes.

A summary of the modifications made for each model is shown in Table 1.

In tissue modelling, each of the AF-1 – AF -4 models also incorporated a reduction to the diffusion coefficient of 40%, to simulate the reduction in the conduction velocity resulting from structural and gap-junctional remodelling (Ausma *et al.* 1997*a*, *b*; van Wagoner *et al.* 1999; van Wagoner & Nerbonne, 2000; Polontchouk *et al.* 2001; van der Velden & Jongsma, 2002; Severs *et al.* 2004, 2008; Bikou *et al.* 2011; for details see Results). For completeness, we have also included work with a model variant (AF-0) in which only the diffusion coefficient was reduced by 60% without considering AF-induced ion channel remodelling. Note that the 60% reduction in the diffusion coefficient in the AF-0 case is greater than that for the AF-1 – AF-4 cases. This is because without considering AF-induced electrical remodelling, at least a 60% reduction in the diffusion coefficient is necessary to sustain re-entry. Otherwise, the same reduction of the diffusion coefficient by 40% as in the AF-1 – AF-4 cases is insufficient to permit sustained re-entry in the tissue model. Note also that Bikou *et al.* (2011) have observed a reduction in connexin protein expression of ∼62% associated with AF, and hence such a reduction can be experimentally justified. In simulations, other forms of the AF-induced structural remodelling (i.e. fibre disorganisation, presence of fibrosis or heterogeneous connexin expression) were not considered.

### Tissue segmentation in the 3D model

The previous 3D anatomical model has originally been based on the Visible Female dataset, and included distinctive regions for the CT, PM, RA and LA (Seemann *et al.* 2006) and also an anatomically accurate description of the sino-atrial node (SAN) (Chandler *et al.* 2011; Aslanidi *et al.* 2011*b*). In this study, we further updated the segmentation of the 3D anatomical model of the human atria by taking into account the presence of other electrophysiologically distinct regions in the atria, such as the AS, AVR, RAA, LAA and PVs. Due to a lack of histological data associated with the Visible Female dataset, these segmentations had to be performed manually, guided by other studies in human (Ho *et al.* 2002; Dössel *et al.* 2012), canine and sheep (Aslanidi *et al.* 2013*b*; Zhao *et al.* 2013). The potential limitations of such an approach are discussed (see Discussion).

### 3D whole atria model

The well known reaction–diffusion equation describes the electrical activity in cardiac tissue (Clayton *et al.* 2011):


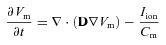
(1)

where *V*_m_ is the membrane potential, ∇ is a 3D spatial gradient operator, **D** is the tensor of diffusion coefficients describing the rate of conduction via gap junctional currents, *I*_ion_ is the total ionic current in a single cell and *C*_m_ is the membrane capacitance. Spatial operators are discretised using a finite differences method, centred differences approach, as described in previous modelling studies (Seemann *et al.* 2006; Aslanidi *et al.* 2011*b*; Colman *et al.* 2011). Equation (1) is then integrated using the forward Euler method. The spatial step used in the integration corresponds to that of the spatial resolution of the 3D anatomical model, 0.33 × 0.33 × 0.33 mm^3^, and the temporal step corresponds to that of the single cell model, 0.005 ms, which guarantee a stable numerical solution of the model (Aslanidi *et al.* 2011*b*). Rule-based fibre anisotropy was included along the bundles of the CT, PM and BB in the atrial model, as in previous models based on the Visible Female dataset (Seemann *et al.* 2006; Aslanidi *et al.* 2011*b*). The anisotropy ratio, describing differences in the intercellular electrical coupling between cells in directions longitudinal and transverse to the fibres, is set to 9:1. This gives experimentally validated conduction velocities along the distinctive fibre bundles of the CT and in the RA wall, which are measured as 1.3 m s^−1^ in the longitudinal direction of the fibre and 0.7 m s^−1^ in the transverse direction of the fibre in the control condition (for more details see Results).

### Analysis of APD distribution and vulnerability windows

Normal AP conduction was initiated in the 3D human atrial model by the application of three consecutive stimuli (with an amplitude of 2 nA and a duration of 2 ms) to the SAN at a basic cycle length (BCL) of 1000 ms. The APD distribution throughout the 3D atrial tissue was mapped by computing the APD_90_ for every computational grid point following the last of three stimuli. The APD was mapped for the control condition as well as for all models of AF-induced remodelling.

The vulnerability of atrial tissue was measured as follows. The S1–S2 pacing protocol was applied to spherical tissue regions of radius 3.3 mm at sites of the CT/PM junction and the LA/PV junction. Ten conditioning stimuli are applied at an S1 cycle length of 350 ms for all models. These were followed by a short coupled S2 stimulus, applied to the same site as the S1 stimuli. The range of S2 coupling intervals for which conduction is permitted in one atrial tissue region but fails in the other is considered the vulnerable window for unidirectional conduction block (VW_CB_). The range of S2 coupling intervals during which a unidirectional conduction block leads to sustained re-entrant circuits (re-entry persisting for more than 4 s; Ridler *et al.* 2011) in that region is considered the vulnerable window for re-entry (VW_R_). By definition, this will necessarily be a subset of the VW_CB_. In some cases, multiple short coupled stimuli were applied to slow the conduction velocity such that sustained re-entry could be permitted – such cases were explicitly identified. The vulnerability window was measured in isotropic tissue to isolate the electrical effects from structural anisotropy effects. This also allowed us to accurately compare the vulnerable window at the CT/PM and LA/PV junctions, as fibre detail was included in the model at the CT/PM junction but not in the LA/PV region.

To quantify the combined impact of alterations to both APD and APD dispersion on tissue vulnerability, we introduced an empirical vulnerability factor to index the likelihood of a functional block at a junction site of two distinctive tissue regions that leads to re-entry:


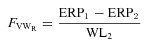
(2)

Where ERP_1_ > ERP_2_ are the effective refractory periods (ERPs) in each of the two regions, and WL_2_ is the excitation wavelength in the region with the shorter ERP. ERP in this case is defined as the minimum S2 coupling interval for which AP conduction occurred. When the S2 stimulus was applied at the junction between the two regions, and the ERP for each region was evaluated in close proximity to the location of the applied stimulus, then ERP_1_– ERP_2_ correlated with the VW_CB_ at that junction. As the VW_CB_ characterises conditions for a unidirectional conduction block, and the wavelength is linked to the minimum size of a re-entrant circuit, the magnitude *F*_VWR_ provides an indication of the likelihood of a unidirectional conduction block leading to sustained re-entry at a particular junction.

### Initiation of atrial fibrillation in 3D atria

Two different protocols for AF initiation in control and AF-remodelled atrial tissue have been considered. In the first protocol, re-entry was initiated by the S1–S2 protocol as described above. The protocol was applied at the CT/PM junction as the larger tissue substrate in the RA is more likely to permit re-entry in control atrial tissue (Aslanidi *et al.* 2011*b*). The S2 coupling interval used in AF simulations was taken at the mid-point of the VW_R_ for each model. Long-term (10 s) AF simulations were performed both with and without the presence of fibre anisotropy.

The second pacing protocol was a series of rapid localised stimuli that mimics long-term pacing of experimental large animal models, which have been demonstrated to initiate AF (Morillo *et al.* 1995; Ausma *et al.* 1997*b*; Sun *et al.* 1998; van Wagoner & Nerbonne, 2000). To simulate such a sustained pacing, all single cell models were first conditioned by stimulating them at a rapid rate (BCL = 140 ms for AF-1 and AF-2, 165 ms for AF-3 and AF-4, and 275 ms for control), and dynamic variables at the time step prior to the final applied stimulus were used to define the initial conditions for the respective regions in the 3D model. Then, 10 stimuli were applied to the 3D tissue model in a region of the LA, at the same rate as in single cells.

### Dominant frequency analysis

Time series of APs at all grid points of the 3D atrial model were recorded and used for Fourier transform analysis, from which the power spectrum was obtained. This was performed for control and various AF models, with the frequency with the largest peak in power spectrum density denoted as the dominant frequency for each case. The dominant frequency was computed using MATLAB. As AF is known to give localised atrial tissue excitation rates between 0.5 and 20 Hz, low (less than 0.1 Hz) and high (larger than 20 Hz) frequency components were eliminated (Hsu *et al.* 2008; Kharche *et al.* 2008). The dominant frequency was defined as the largest peak frequency of the remaining power spectrum. Any peaks that were not harmonics of the dominant frequency were also taken into account in our analysis and interpretation of the results.

## Results

### Single cell model

**Figure 1:**
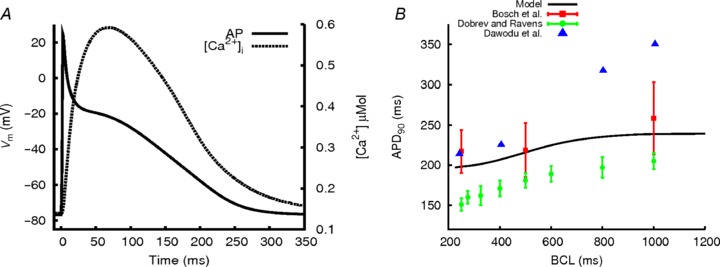
Single atrial cell action potential, Ca^2+^ transient and APD restitution *A*, the AP produced by the modified CRN cell model, simulated at a BCL of 1000 ms (continuous line) and the intracellular Ca^2+^ concentration change (Ca^2+^ transient) during the AP trace (dotted line). *B*, APD restitution for the model, compared to available experimental data (Dawodu *et al.* 1996; Bosch *et al.* 1999; Dobrev & Ravens, 2003).

Figure 1*A* shows the computed AP from the updated CRN model and the intracellular Ca^2+^ transient during the AP time course. Compared to the original CRN model, the updated model preserves the characteristic spike and dome morphology of the human atrial AP, but with a shorter AP duration (APD_90_ is 242 ms at a BCL of 1000 ms, compared to 297 ms of the CRN model, Online Supplement Figure S2) that closely matches experimental data (Dawodu *et al.* 1996; Bosch *et al.* 1999). Note that due to the inclusion of new formulations of *I*_to_ and *I*_Kur_ and Ca^2+^ handling system, the AP has a less pronounced dome, a longer time course of phase 1 repolarisation, and the mean Ca^2+^ transient (averaged from peripheral and interior sites of the cytoplasmic space) has a slower two-phase upstroke and a more rounded peak that is similar to that of the KM model. The computed APD restitution curve of the updated model was also found to match closely published experimental data (Fig. 1*B*; Dawodu *et al.* 1996; Bosch *et al.* 1999; Dobrev & Ravens, 2003). The model also exhibits an improved long-term stability of the solutions on the ionic concentrations of the original CRN model (Online Supplement Fig. S2E). These differences collectively represent a significant improvement to the CRN model. A detailed comparison of the updated model with experimental data is provided in the Online Supplement (Fig. S3, Table S1).

### Regional cell models

**Figure 2:**
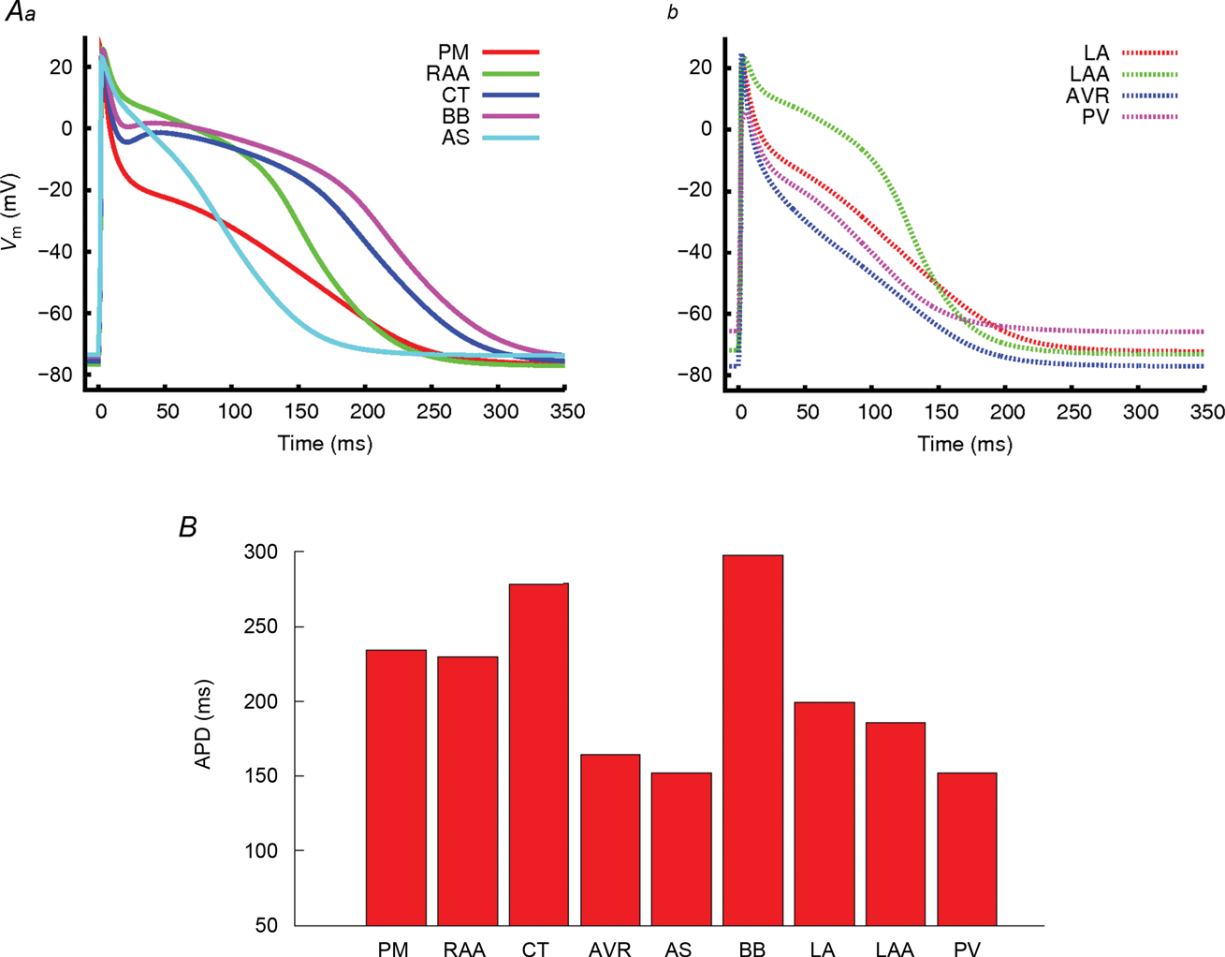
APs computed from regional atrial cell models and their APDs *A*, AP traces for the regional cell models for (*a*) the pectinate muscle (PM, solid red), right atrial appendage (RAA, solid green), crista terminalis (CT, solid blue), Bachmann's bundle (BB, solid pink) and atrial septum (AS, solid light blue), and (*b*) left atrial wall (LA, dotted red), left atrial appendage (LAA, dotted green), atrio-venrticular ring (AVR, dotted blue) and the pulmonary veins (PV, dotted pink). *B*, plots of APD_90_ for each of the different regional cell models measured at a BCL of 1000 ms.

APs simulated with the family of regional cell models are shown in Fig. 2*A*, and the APD_90_ measured for each model at a BCL of 1000 ms is shown in Fig. 2*B*. A high degree of variation in AP morphology and APD between different cell types was found: BB and CT had the longest APD, almost double that of the AVR, PVs and AS, with the AS displaying the shortest APD (e.g. APD_90_ in the BB is 297 ms, compared to 152 ms in the AS). There was also a large variation in the notch and plateau potential, as well as in the resting potential among the different cell types. All these characteristic differences between the different cell models agree well with available experimental data from human or dog, which are summarized in Table 2. Note that the RAA model was validated using experimental data from human (APD_90_ in the model is 230 ms at a BCL of 1000 ms, compared to 255 ± 45 ms observed by Bosch *et al*. (1999)) and other models were validated by their APD relative to the RAA using either human or canine data (as necessitated by human data availability or lack thereof; Feng *et al.* 1998; Li *et al.* 2001; Burashnikov *et al.* 2004; Katoh *et al.* 2005; Gong *et al.* 2008). Additional details of the AP morphology validation are presented in the Online Supplement (Figs S8–S12). Notably, significant regional APD heterogeneity was observed at both fast and slow rates (results are shown in Supplemental Fig. S13).

**Table 2:** Regional proportional APD_90_ properties in the models and experiment

Region	Model	Experimental values
CT with respect to RAA	1.21	1.28 (Katoh *et al.* 2005),
		1.12 (Burashnikov *et al.* 2004),
		1.41 (Feng *et al.* 1998) (APD_95_)
AVR with respect to RAA	0.71	0.89 (Feng *et al.* 1998) (APD_95_)
AS with respect to RAA	0.66	0.71 (Gong *et al.* 2008)
BB with respect to CT	1.07	0.96–1.07 (Burashnikov *et al.* 2004)
LA with respect to RAA	0.86	0.89 (Li *et al.* 2001)
PM with respect to RAA	1.03	1.05 (Feng *et al.* 1998) (APD_95_)

### Effects of AF-induced electrical remodelling at the single cell level

**Figure 3:**
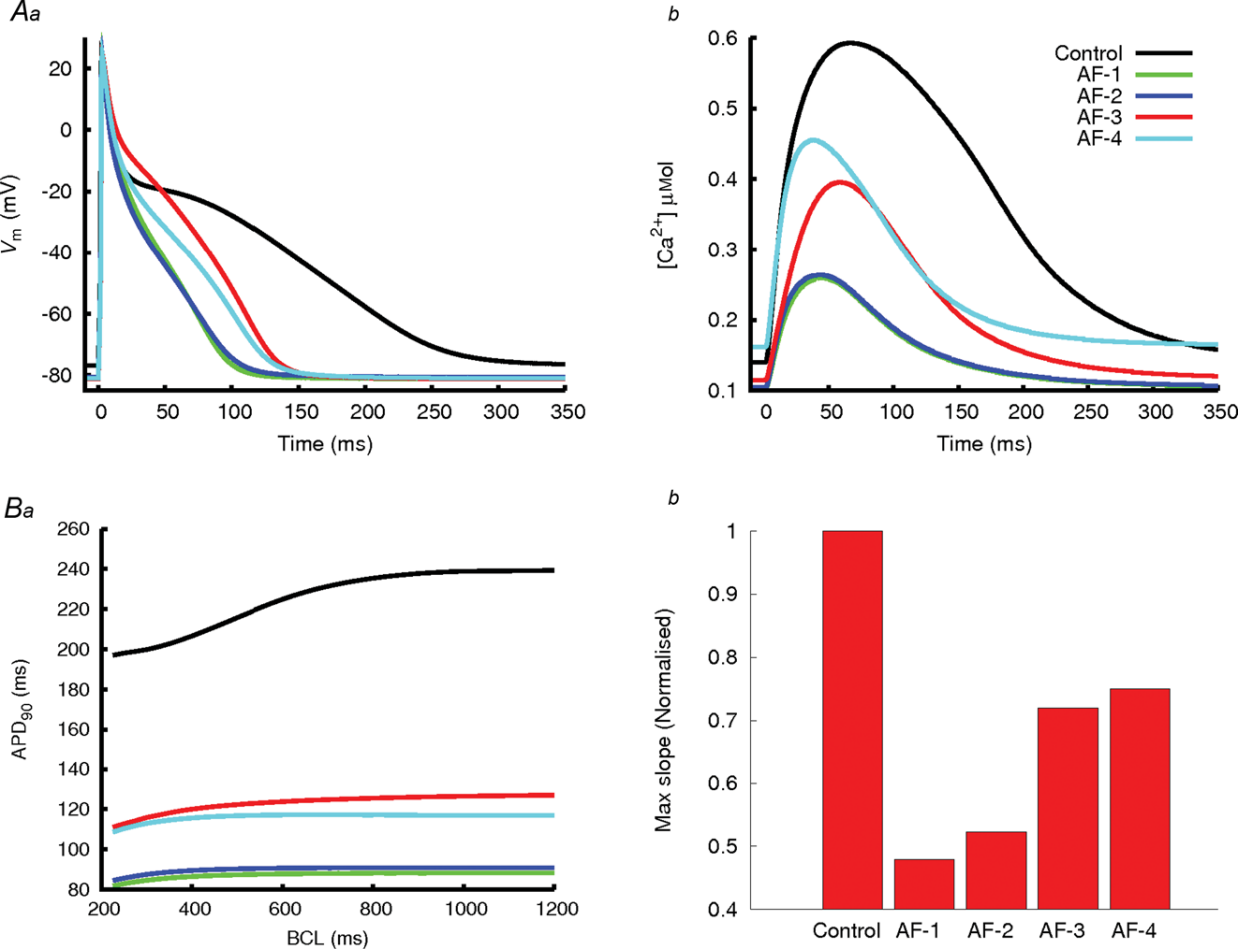
Effects of AF-induced remodelling on single atrial cell AP, [Ca^2+^]_i_ and APD restitution *A*, APs (*a*) and Ca^2+^ transient (*b*) in control (black lines) and the four AF-induced remodelling models (coloured lines). *B*, APD restitution in control and AF-induced remodelling models (*a*); and maximum slope of APD restitution for control and AF conditions (*b*). The slope in control has been normalized to 1 and then each AF condition expressed as a fraction of control.

The effects of four scenarios of AF-induced electrical remodelling on single atrial cell APs are shown in Fig. 3*A* along with the control model. In each of the AF-1 – AF-4 cases, the AF-induced electrical remodelling produced a reduction of the APD and more triangular APs (Fig. 3*Aa*), as well as a significant reduction in the magnitude and duration of the Ca^2+^ transient (Fig. 3*Ab*). Cases AF-1 and AF-2, which do not include reductions to *I*_Kur_, result in greater APD shortening and more significantly reduced the Ca^2+^ transient, in comparison to AF-3 and AF-4, where remodelling to *I*_Kur_ was included. When AF-induced remodelling to the subcellular Ca^2+^ handling processes was included (AF-4), an elevation to the diastolic and systolic Ca^2+^ levels was observed (Fig. 3*Ab*), which matches experimental observations (Neef *et al.* 2010; Voigt *et al.* 2012).

The simulated APD reduction in each AF case agrees with experimental data. In simulations, AF-1 resulted in a 62% reduction in APD_90_, which is comparable to the 60% observed by Bosch *et al.* (1999); AF-2 resulted in a 60% reduction in APD_90_, which is close to the 55% reduction observed by Workman *et al*. (2001); AF-3 resulted in a 45% reduction in APD_90_, comparable to the 44% reduction observed by van Wagoner *et al.* (1999); and AF-4 resulted in a 49% reduction in APD_90_, which lies within the large experimental range of 23–60% (Bosch *et al.* 1999; Dobrev & Ravens, 2003). All four models resulted in a reduction of the rate dependency of the APD (Fig. 3*Ba*), as well as a reduction of the maximum slope of the restitution curve (Fig. 3*Bb*), in agreement with experimental data (van Wagoner *et al.* 1999; Bosch *et al.* 1999; Dobrev & Ravens, 2003). Such a loss of APD rate adaption has been identified in AF-remodelled atrial cells (van Wagoner *et al.* 1999; Bosch *et al.* 1999; Dobrev & Ravens, 2003).

**Figure 4:**
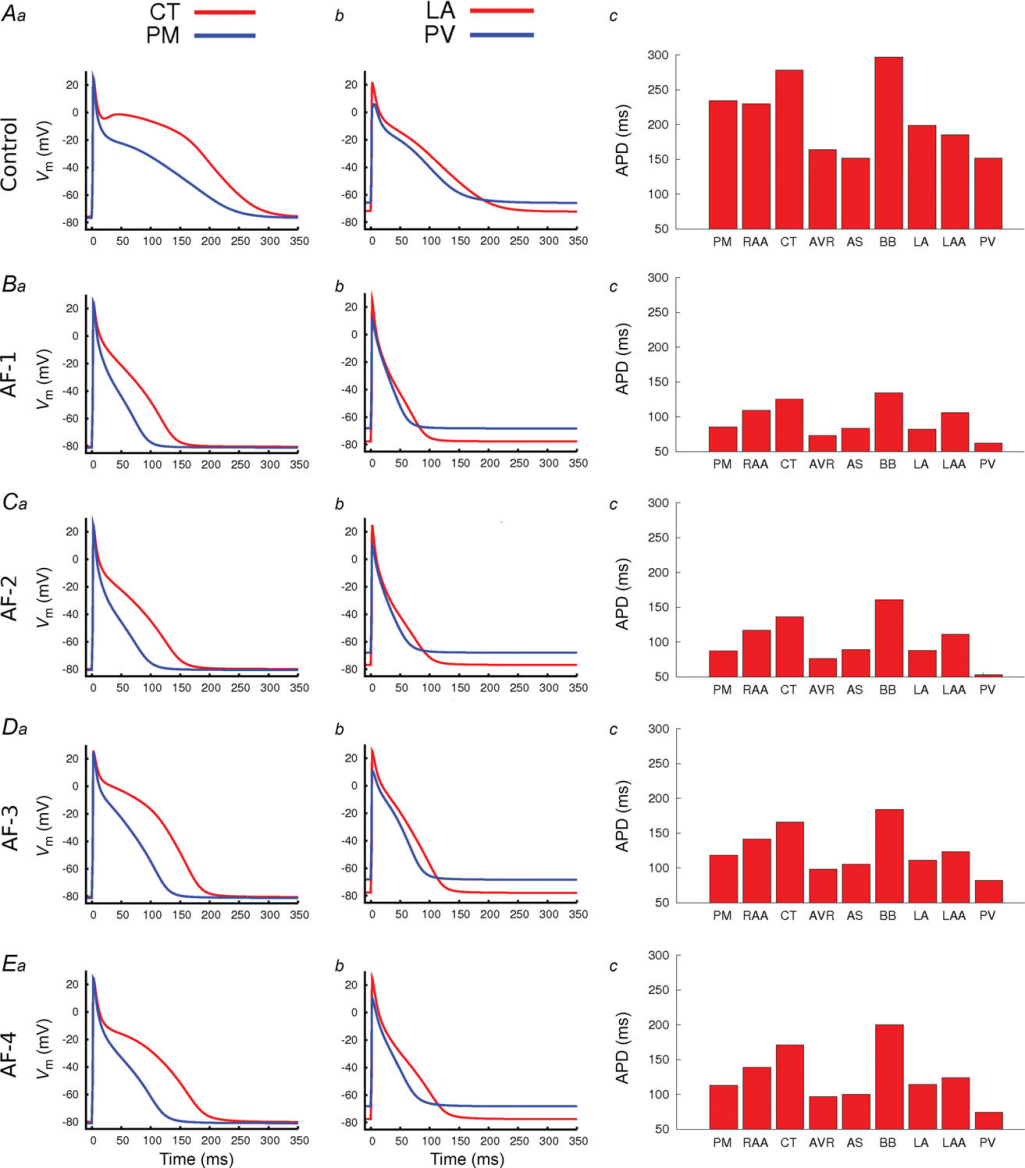
Effect of AF-induced remodelling on AP regional heterogeneity Comparisons were made at the junctions of the CT/PM and LA/PV. For each panel, APs are overlain for the CT and PM (*a*), LA and PV (*b*), whilst APD_90_ for all regional cell models are shown in *c*. *A*, data in control; *B–E*, data for the different AF-induced remodelling conditions (as indicated on the left hand side of *a* in each row).

The effect of AF-induced electrical remodelling on regional heterogeneity in single atrial cells is shown in Fig. 4. This shows, for control and AF conditions, AP traces from the CT/PM and LA/PV cell types (the two major junctions with dramatic regional heterogeneity) (Fig. 4*a,b*) and APD from all cell types (Fig. 4*c*). It was observed that the AF-induced electrical remodelling had a substantial effect on the atrial heterogeneity: the APD in all cell types was shortened, but APD differences at the CT/PM and LA/PV junctions remain (compare Fig. 4*Aa*, *b* with Fig. 4*Ba*, *b* to *Ea*, *b*). For example, the difference in APD_90_ between the LA and PV is 47 ms in control, compared to 40 ms in AF-4 and 20 ms in AF-1. Therefore, although the electrical remodelling abbreviated APD in all cell types, APD dispersion was preserved (Fig. 4*c*). Note that among the four AF remodelling cases, AF-1 and AF-2 result in a greater APD heterogeneity reduction as compared to AF-3 and AF-4. The observed cellular APD heterogeneity was little affected by cycle length due to reduction in rate adaption of APD under remodelling (i.e. flattened APD restitution curves).

### Activation of 3D atrial model

**Figure 5:**
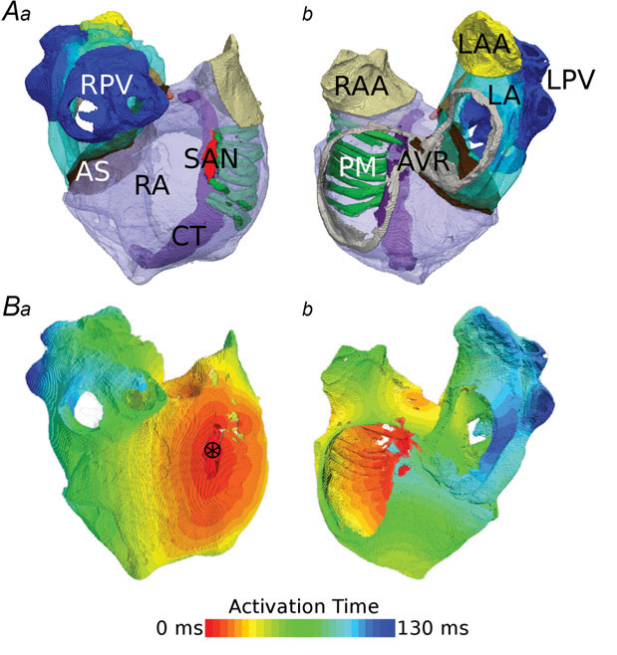
3D anatomical model *A*, fully segmented atria from two different views (*a*– looking at the RA wall, *b*– view into the atrial cavities), with the RA in transparent purple, CT in solid purple, PMs in green, SAN in red, AS in brown, LA in transparent light blue, PVs in solid dark blue, RAA in beige, LAA in yellow and AVR in grey. All regions are labelled. *B*, normal activation in the fully segmented model from the same views. Please note this has been paced from the SAN region, indicated by the asterisk in *a*.

Figure 5 shows the segmented 3D atrial anatomical model (Fig. 5*A*) and the simulated atrial activation sequence, following an applied stimulus to the SAN (Fig. 5*B*). In the control condition, the total activation time was 130 ms, in agreement with experimental observations by Lemery *et al.* (2007). Modification of the CRN single cell model and introduction of a large degree of regional heterogeneity had no significant effect on the atrial activation sequence compared to our previous model (Aslanidi *et al.* 2011*b*). Conduction velocities were measured as 0.7 m s^−1^ transverse to atrial fibres in the RA wall and 1.3 m s^−1^ longitudinal to the fibres in the CT, in agreement with experimental values (Boineau *et al.* 1988; Lemery *et al.* 2007; Fedorov *et al.* 2011) and our previous simulation results (Aslanidi *et al.* 2011*b*). The reduction in the diffusion coefficients by 40% alone (employed as part of AF-induced remodelling models; see Methods) results in a reduction of the conduction velocities to 0.54 m s^−1^ in the RA wall and 1.0 m s^−1^ longitudinal to the fibres in the CT.

### Effects of AF remodelling on APD dispersion in the 3D atria

**Figure 6:**
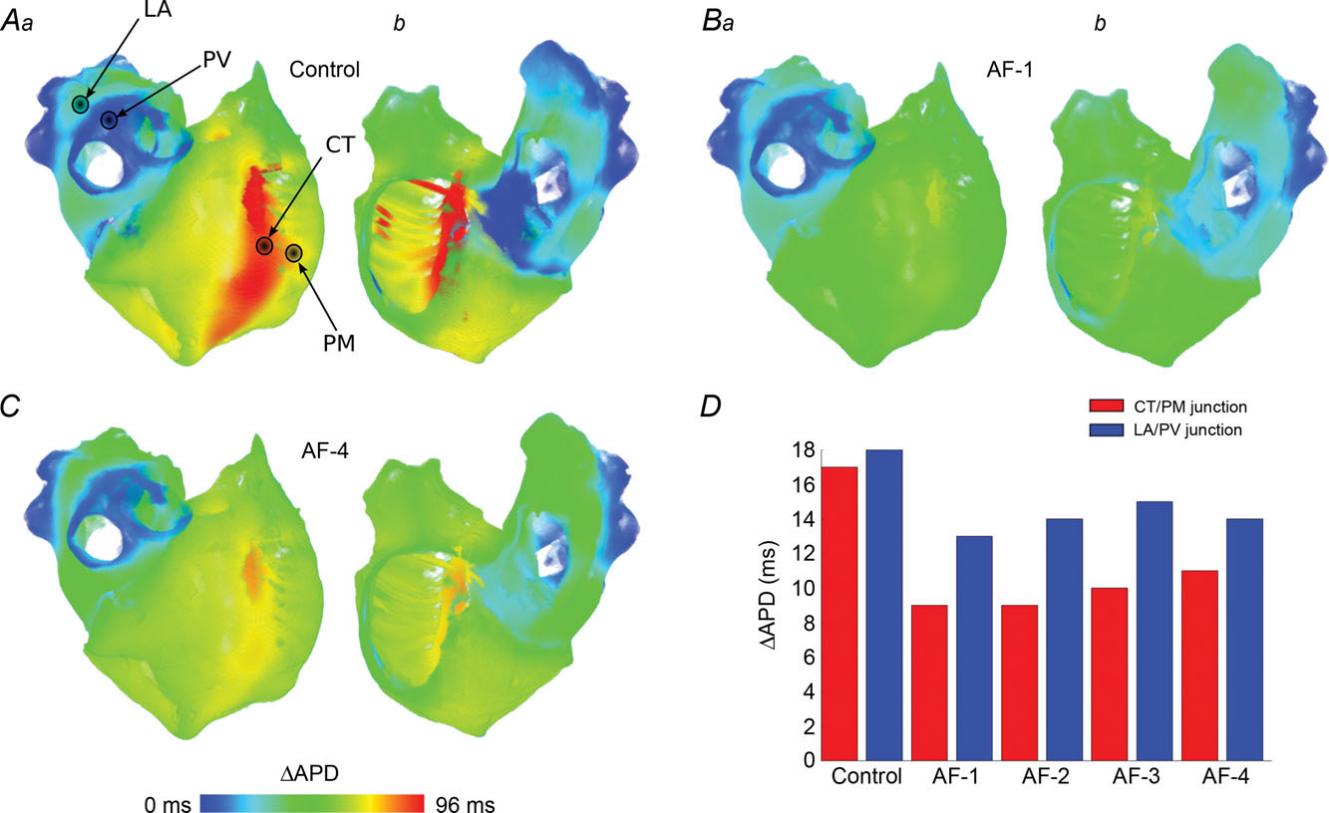
Spatial change in APD distribution in the whole 3D atria in control and with AF-induced remodelling *A–C*, ΔAPD distribution maps for control (*A*), AF-1 (*B*) and AF-4 (*C*). Colours are scaled such that ΔAPD = 0 ms is the shortest APD in tissue in each. Hence, the relative distribution of APD is illustrated in these figures. *D*, the difference in APD between the CT and PM (red) and the LA and PV (blue) in tissue in each model. AP recording sites for each region are indicated in *A*.

The dispersion of APD_90_ in the whole atria following normal pacing is shown in Fig. 6. In control (Fig. 6*A*), the heterogeneity observed in single cells is largely preserved in the tissue. Electrotonic effects due to cell-to-cell coupling reduce the maximum difference in APD_90_ between the AS and BB from 145 ms in a single cell to 96 ms in tissue, but at the junctions of the CT and PM in the RA and the PV and LA the large APD gradient is preserved. AP recordings from single locations within each of these regions close to the junctions (Fig. 6*A*) show the difference in APD_90_ between the CT and PM cells of 17 ms, and between the LA and PV cells of 18 ms (Fig. 6*D*).

As in the case of single cells (Fig. 4), AF-induced remodelling reduces but does not eliminate regional APD heterogeneity in the 3D atria (Fig. 6*B* and *C*), where the APD gradients are preserved at the CT/PM and LA/PV junctions (Fig. 6*D*). Note that heterogeneity at the LA/PV junction is preserved to a greater degree than at the CT/PM junction, which may have important implications for the vulnerability to re-entry. Also similarly to single cells (Fig. 4), AF-1 and AF-2 result in the largest reduction of regional heterogeneity: AF-1 reduces maximum APD dispersion to 52 ms, compared to AF-4 which reduces APD dispersion to 80 ms, from 96 ms in control.

### Vulnerability to unidirectional conduction block and re-entry

**Figure 7:**
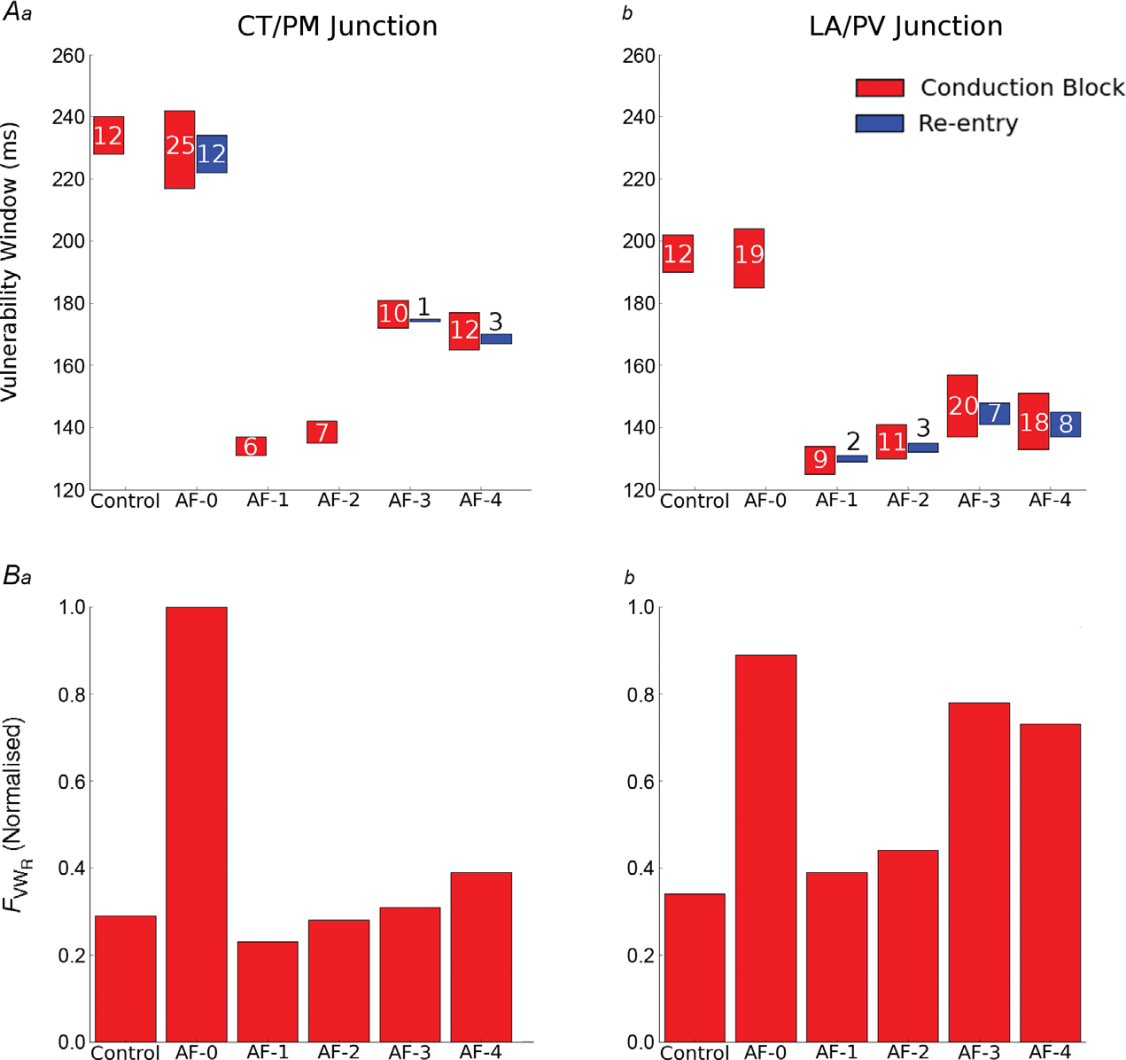
Measured time window for unidirectional conduction block and re-entry *A*, time window of vulnerability measured in the 3D human atria at the CT/PM junction (*a*) and LA/PV junction (*b*). The time window for unidirectional conduction block is shown in red and sustained re-entry in blue. Where there is no blue box, sustained re-entry could not be initiated. The width of the window (ms) is labelled by the numbers within or above each box. *B*, magnitude of *F*_VWR_at each of the two regions is plotted for each condition. Magnitudes are normalised to the value obtained in AF-0 at the CT/PM junction.

The VW_CB_ at the two regional junctions in the 3D human atria for control and AF-induced remodelling conditions are shown in Fig. 7*A*. Electrical remodelling shifts the VW_CB_ towards shorter coupling intervals and also reduces the extent of the windows. AF remodelling conditions that result in the greatest APD_90_ reduction (AF-1 and AF-2) are characterised by the smallest VW_CB_. In all remodelling cases, the measured VW_CB_ are greater at the LA/PV junction than at the CT/PM junction. Note that the VW_CB_ for AF-0 is larger than that observed in control due to the reduction in the electrotonic interaction as a consequence of reduced cell-to-cell coupling.

The measured VW_R_ are also shown in Fig. 7*A*. In simulations, sustained re-entry can only be initiated at the CT/PM junction following the application of multiple S2 stimuli (see Methods), whereas at the LA/PV junction one S2 stimulus is sufficient. Sustained re-entry could not be initiated at either junction in control, but could be initiated at the CT/PM junction in AF-0.

The largest VW_R_ at the CT/PM junction occurs in the AF-0 condition, where large VW_CB_ promoting the initiation of re-entry is combined with a short wavelength of atrial excitation (compared to the size of the RA), enabling the tissue to sustain re-entry. Sustained re-entry can also be initiated in AF-3 and AF-4 within a small VW_R_, but cannot be initiated in AF-1 and AF-2 (in these cases, re-entry self-terminated after fewer than three cycles). At the LA/PV junction, re-entry cannot be initiated in control or AF-0 conditions because of the long wavelength of atrial excitation compared to the small LA substrate in the model, but can be initiated in all electrical remodelled conditions (AF-1 – AF-4) as the wavelength is decreased. In all AF remodelling cases, the measured VW_R_ is greater at the LA/PV junction than at the CT/PM junction (Fig. 7*A*).

### Mechanism of re-entry initiation

**Figure 8:**
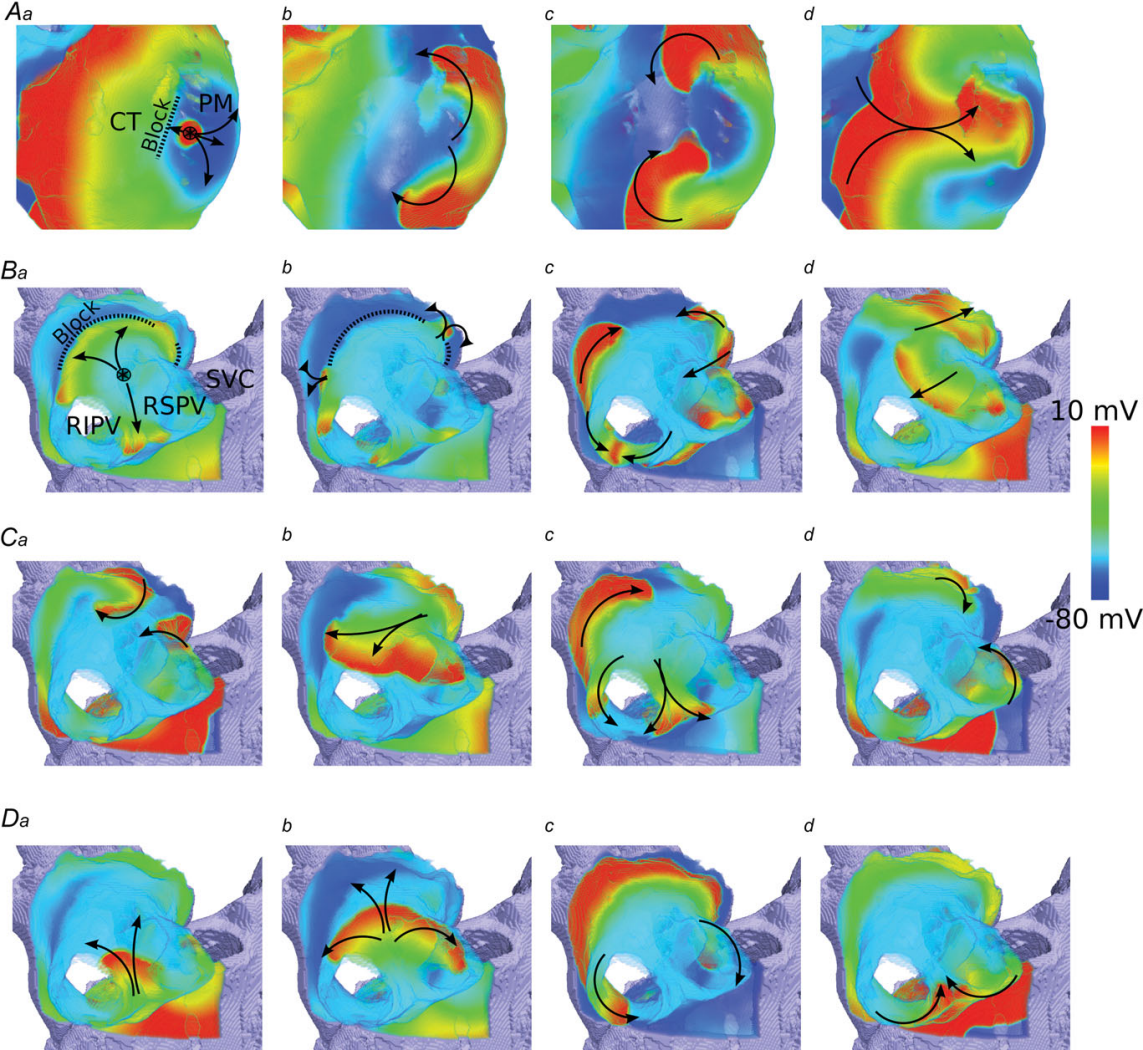
Initiation of re-entry by S1–S2 protocol *A*, snapshots of initiated re-entry at the CT/PM junction at a time after the S2 stimulus: *t*= 5 ms (*a*), 70 ms (*b*), 115 ms (*c*) and 175 ms (*d*). *B*, snapshots of initiated re-entry in the PVs at a time after the S2 stimulus: *t*= 35 ms (*a*), 55 ms (*b*), 70 ms (*c*) and 190 ms (*d*). *C* and *D*, two illustrations of final re-entrant pathways emerging in the PV region. Distribution of the membrane potential in the 3D atrial tissue is shown using a standard rainbow palette. Dotted line means functional conduction block. Arrows indicate wave propagation. Circle with an asterisk is the location of the applied stimulus.

To elucidate the mechanisms underlying the initiation of re-entry in different regions of the 3D atria, we further analysed the AP conduction pattern following S1–S2 pacing. Results are shown in Fig. 8 and Videos S1–S3 in the Online Supplement. At the CT/PM junction (Video S1), the short coupled S2 stimulus permitted conduction in the PM and RA, but not towards the CT (Fig. 8*Aa*), due to its longer APD in comparison with the PM and RA (Fig. 2*B*). This conduction block in the CT region is due to the incomplete recovery of the tissue from the previous excitation evoked by the S1 stimulus. After some time the CT repolarised, and the excitation wave was then able to propagate back into this region (Fig. 8*Ab*,*c*), eventually returning into the PM region and completing a full re-entrant circuit (Fig. 8*d*).

In the PV region, the conduction pattern in response to the S2 stimulus was affected by the complex anatomical structure of this region. In simulations, different conduction pathways of the excitation wave were observed for control and the various AF conditions, including differences in the break-through points of the excitation wave into the LA, the re-entry points into the PVs and the degree of break-down within the PVs themselves. The general excitation pattern common to all AF simulations of the PV region in response to an S2 stimulus (coupling interval of 145 ms) is illustrated in Fig. 8*B* and Video S2 (for the AF-4 case). The excitation wavefront was initially blocked from entering the LA (Fig. 8*Ba*) due to the longer APD in the latter region. After some time when the LA had recovered its excitability following the previous S1 excitation, the S2 excitation wave entered the LA region (Fig. 8*Bb*); however, the single wavefront broke down, forming multiple wavefronts. Some of these wavelets were annihilated (Fig. 8*Bc*) while others persisted, to drive excitation (Fig. 8*Bd*). Through this mechanism of breakdown and annihilation, eventually a pair of excitation wavefronts remained. All AF simulations considered in this study resulted in one of two final propagation patterns: a pair of rotors meandering around the PVs and the PV/LA junction (Fig. 8*C*, Video S2), and a rigid pattern of rotation around the right PVs (RPVs; Fig. 8*D*, Video S3).

In both the CT/PM and the PV/LA junctions, and in all AF remodelling and control conditions, the general mechanisms by which sustained re-entry was initiated by the short coupled S1–S2 stimulus is similar: a unidirectional conduction block towards the atrial tissue region with longer APD/ERP resulted in breakdown of a regular excitation wave, which could develop into sustained re-entry.

### Atrial fibrillation by rapid focal pacing

**Figure 9:**
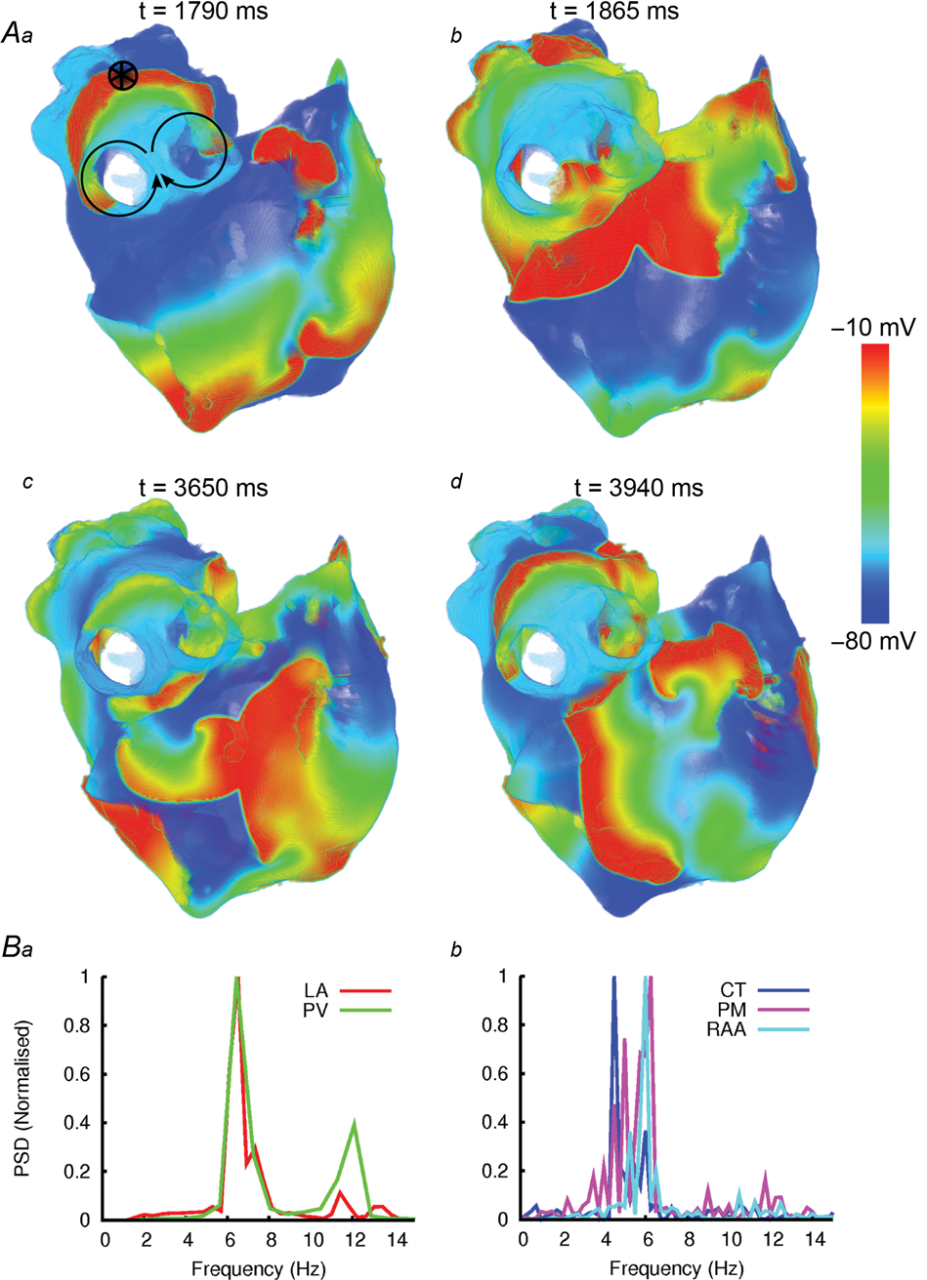
Simulated AF induced by rapid pacing *A*, snapshots of wave propagation at various timings (*a*–*d*), illustrating the development of a re-entrant circuit around the RPV (*a*, *b*) and the development and breakdown of re-entry in the RA (*c*, *d*). The distribution of the membrane potential in the 3D atrial tissue is shown using a standard rainbow palette. Circle with an asterisk indicates the stimulation site. *B*, dominant frequency analysis of locations within the LA (*a*) and RA (*b*).

We further investigated the generation of re-entry in response to a sequence of rapid atrial focal stimuli near the PV region. In simulations, sustained re-entry was successfully initiated by using the rapid pacing protocol in all AF-1 – AF-4 models, but this did not occur in control or AF-0 (re-entry self-terminated in both cases). The mechanism by which re-entry was formed is similar in all AF cases, illustrated in Fig. 9 and Video S4 in Online Supplement for the case of AF-4. Shortly after the last applied stimulus, the excitation wave broke down at the junction of the LA and PVs, developing into a re-entrant wave which rotated around the RPV (the same circuit as illustrated in Fig. 8*D*). This re-entry continued to rotate for the full duration of the simulation (10 s), with a cycle length of 162 ms for AF-4. In AF-3 and AF-4, excitation waves emitted from such a rapid rotor in the PV broke down in the RA to form multiple re-entrant circuits (Fig. 9*Ab–d*). In AF-1 and AF-2, the waves did not break down sufficiently in the RA to develop into new re-entrant circuits. Figure 9*B* shows the results of dominant frequency analysis of the case for AF-4. The highest dominant frequency is seen in the PV and LA region, followed by the PM and RAA regions, with the CT having the lowest frequency. Frequency variance in the RA is also significantly larger than in the LA due to the presence of multiple re-entrant wavelets. Dominant frequency analysis of AF-3 yielded similar results. In the cases of AF-1 and AF-2, the frequency variance in the RA was similar to that observed in the LA, due to the absence of self-sustained re-entrant circuits in this region. Hence, sustained rapid pacing can develop into re-entry by similar mechanisms as elicited by an S1–S2 pacing protocol.

### Long-term behaviour following S1–S2 stimulation

**Figure 10:**
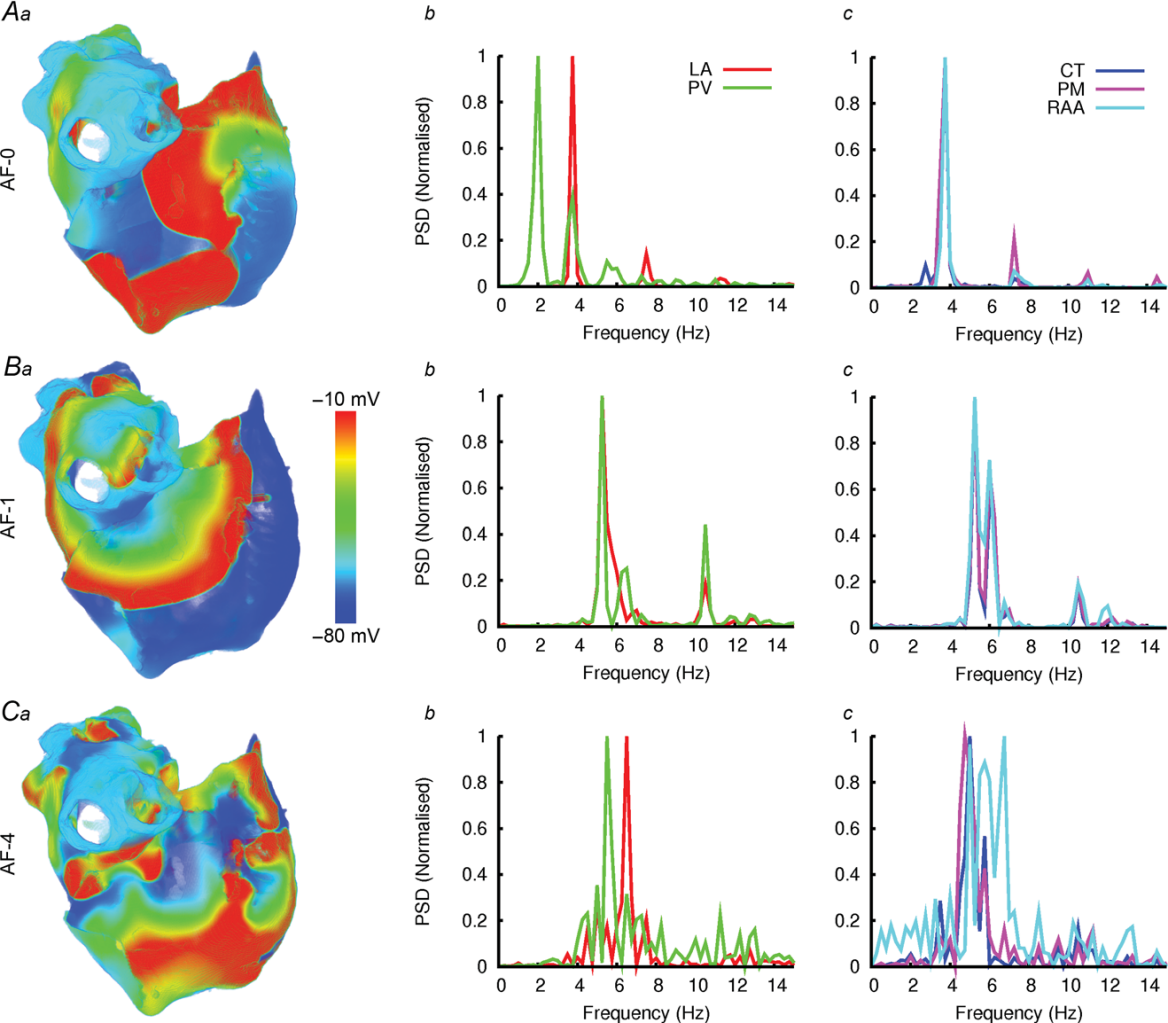
Comparison of AF between control and AF remodelled conditions *A–C*, comparison of AF between control (*A*) and AF remodelled (*B*, *C*) conditions. The considered AF cases are indicated in the respective panels. *a*, illustration of re-entrant excitation waves for control and AF conditions; *b* and *c*, dominant frequency analysis in different regions.

AF was successfully initiated in models AF-0 – AF-4 using the S1–S2 protocol. The long-term behaviour of such re-entry varied between the different AF models. In AF-0, AF-3 and AF-4 simulations re-entry was sustained in the RA. In AF-0, the initiated re-entrant excitation wave meandered to a small extent around the RA and then broke down to form a few multiple re-entrant wavelets (Fig. 10*A*, Video S5 in Online Supplement). In AF-3 and AF-4, the wave initially meandered to a much greater extent, before breaking down in the RA to form many multiple re-entrant wavelets (Fig. 10*C*, Video S6 in Online Supplement), and in the LA to form a single rotor wave around the PVs or base of the LAA.

However, the initiated re-entry could not be sustained in the RA in the case of AF-1 and AF-2. In both cases, two complete re-entrant circuits were permitted in the RA before self-termination. Within this time period, the wave spread into the LA and broke down into a rotor propagating around the RPVs. The wave circulating around the RPVs became a driving rotor with a high frequency, and therefore controlled atrial excitation. This driving rotor sustained throughout the duration of the simulation (10 s), with no further breakdown observed in the RA (Fig. 10*B*, Video S7 in Online Supplement). The dominant frequency analysis showed that AF-4 (and also AF-3, not shown) exhibited the greatest degree of frequency variance, associated with multiple re-entrant wavelets, and that all conditions (AF-1 – AF-4) permitted significantly higher frequencies than AF-0.

## Discussion

In this study, we have updated the 3D virtual human atria (Aslanidi *et al.* 2011*b*) by incorporating multiple data sets to simulate AF-induced electrical remodelling. The newly developed model has been used to explore the effects of remodelling on electrical heterogeneity and vulnerability to re-entry in the 3D atria. Our major findings are as follows. (1) In all AF-1 – AF-4 cases, the AF-induced electrical remodelling abbreviated atrial APD. Such APD reduction was heterogeneous across the atria, resulting in a reduced but preserved regional heterogeneity. Importantly, large APD gradients remained at the junctions of the CT with the PM and the LA with the PV. (ii) AF-induced electrical remodelling increased tissue vulnerability to initiation of re-entry at these tissue junctional regions. As the APD gradients were preserved by a larger degree at the LA/PV junction than the CT/PM junction in remodelled cases, the measured vulnerability in the LA/PV region was greater than that in the CT/PM region. (3) The AF-induced electrical remodelling also stabilized and accelerated re-entrant excitation waves, leading to rapid and sustained re-entry. (4) Regional APD heterogeneity promoted the degeneration of re-entrant waves into persistent erratic wavelets in the electrically remodelled atria. These findings collectively demonstrate that the AF-induced electrical remodelling increases atrial susceptibility to arrhythmia due to increased tissue vulnerability and shortened APD, which respectively facilitate initiation and maintenance of re-entrant excitation waves. They also provide new insights into the complex mechanisms of clinical AF and the significant role of the PVs in such behaviour.

### Comparison with experimental data

At the cellular level, all of the AF remodelling conditions considered here led to abbreviated atrial APD, in quantitative agreement with various experimental data (van Wagoner *et al.* 1997, 1999; Bosch *et al.* 1999; Workman *et al.* 2001) on which each of the AF remodelling conditions was based. At the 3D tissue level, the simulated atrial activation patterns (Fig. 5) and atrial activation time also matched experimental data (Lemery *et al.* 2007). Under AF remodelling conditions, the atrial cellular APD was non-uniformly abbreviated. This is consistent with an experimental animal study of Fareh *et al.* (1998), which demonstrated that heterogeneously remodelled atrial refractoriness played an important role in increasing atrial vulnerability to AF induction and the duration of induced AF in dogs.

Using the 3D model we have simulated the initiation and maintenance of re-entrant excitation waves, and how these sustain and then degenerate into multiple wavelets in control and electrically remodelled atria. Our simulation data in this regard also match experimental data.

Experimental and clinical evidence have suggested that: (1) atrial tissues with large regional differences in electrical properties are more susceptible to re-entry (Allessie *et al.* 1976; Spach *et al.* 1989); (2) re-entrant wavelets propagating in an abnormal atrial tissue substrate can sustain high-frequency irregular electrical activity in AF (Harada *et al.* 1996; Jalife *et al.* 2002; Nattel *et al.* 2005); (3) ‘driver’ regions acting as the dominant frequency sources during AF may be located in the LA near the PV sleeves, whereas the RA is characterised by multiple wavelets (Haïssaguerre *et al.* 1998; Chen *et al.* 1999; Sanders *et al.* 2005); (4) rapid AF is also often progressive, going from paroxysmal to chronic, with electrical and structural remodelling of the atria leading to a substrate that facilitates self-perpetuation of the arrhythmia (Nattel *et al.* 2005; Anter *et al.* 2009).

Our modelling results are in accord with these experimental observations: (1) large APD gradients at the CT/PM and PV/LA junctions (Fig. 6) resulted in high vulnerability to re-entry (Fig. 7); (2) break down of electrical excitation waves at the junctions led to the generation of re-entrant waves characteristic of AF (Fig. 8); (3) there was a single dominant frequency rotor in the PV region of the LA, and multiple re-entrant wavelets in the RA (Fig. 9); (4) AF-induced electrical remodelling increased the dominant frequency and overall perpetuation of AF (Fig. 10).

Note that multiple re-entrant wavelets were produced in the 3D simulations by rapid atrial pacing. This stimulation protocol used is the same as that commonly used in animal experimental settings for initiating AF (Cox *et al.* 1991; Mandapati *et al.* 2000). Clinical studies have observed high-frequency sources of electrical activity near the PV sleeves in AF patients (Haïssaguerre *et al.* 1998; Sanders *et al.* 2005), with a cycle length of ∼170 ms (and, hence, frequency of ∼6 Hz). Our simulation data on the high-frequency driving rotor inthe PV region match closely these clinical observations, as the computed cycle length of re-entrant excitation wave in our simulations was ∼162 ms (i.e. ∼6.1 Hz) (see AF-4 model in Fig. 10).

### Effect of remodelling on vulnerability to re-entry

This study has shown that regional APD heterogeneity is reduced but not eliminated under electrical remodelling conditions both in single cells and at the level of 3D atrial tissue. Such reduced APD gradients are seen at the junctions of the CT/PM and LA/PV, with a greater preserved gradient at the LA/PV than the CT/PM junction. Whereas shorter APDs facilitate the maintenance of re-entry (Pandit *et al.* 2005), the reduction in APD dispersion is expected to reduce the likelihood of a unidirectional conduction block leading to re-entry. The combined impact of shortened APD and reduced APD dispersion on initiation and maintenance of re-entry can be characterised by *F*_VWR_ (eqn 2) as discussed below.

There is a strong correlation between the magnitude of *F*_VWR_ and the VW_R_ in each region in AF-1 – AF-4 remodelling conditions (Fig. 7*B*). Note that this factor does not account for the substrate size, and therefore is not applicable in control or AF-0, for which the substrate is too small (compared to the long non-remodelled wavelength) to permit sustained re-entry. This factor is largest in the LA/PV region due to a large APD/ERP gradient between them and a short wavelength in the PV region. This may explain why this region has a high vulnerability to re-entry: re-entrant circuits developed in the LA/PV region in all remodelling conditions, and with all pacing protocols used (Fig. 7).

### Comparison with other models

Biophysically detailed 3D computational models have been developed to account for various aspects of atrial electrophysiology and anatomy (Seemann *et al.* 2006; Kharche & Zhang, 2008; Aslanidi *et al.* 2011*b*; Dössel *et al.* 2012; Kharche *et al.* 2012; McDowell *et al.* 2012). However, most models have not accounted for the electrical heterogeneity of the atria and its changes due to AF-induced electrical remodelling. Our recent 3D human atrial model (Aslanidi *et al.* 2011*b*), which incorporated detailed electrophysiological heterogeneity, has been updated here to account for (1) additional experimental data and (2) the effects of AF-induced electrical remodelling.

Primarily, new formulations of some of the outward K^+^ currents (*I*_to_ and *I*_Kur_; Maleckar *et al.* 2009) and the intracellular Ca^2+^ handling system (Koivumäki *et al.* 2011) were incorporated into the CRN atrial cell model. The updated CRN cell model showed better correspondence with experimental data (van Wagoner *et al.* 1999; Bosch *et al.* 1999; Workman *et al.* 2009), while preserving its basic features and suitability as a base model for the development of regional cell models (Figs 1 and 2). Incorporation of the new intracellular Ca^2+^ handling system also significantly improved the long-term ionic stability of the CRN model, which has been identified as one of the primary limitations of the CRN model (Wilhelms *et al.* 2012). The updated regional cell models also demonstrated a good agreement to the previous models (Seemann *et al.* 2006; Aslanidi *et al*. 2011*b*; Dorn *et al.* 2012). The difference from the previous models was primarily in terms of a better agreement of AP morphology in the RAA model with the available data (Gong *et al.* 2008). Most previous models (that by Dorn *et al.* (2012) being a notable exception) have not considered distinct cell models for the BB and PV, which were added in the present study. The PV electrophysiology model in particular may be crucial in understanding the mechanisms of high-frequency electrical activity near the PV sleeves, which has been strongly linked with the genesis of AF (Haïssaguerre *et al.* 1998; Sanders *et al.* 2005; Calkins *et al.* 2012).

Development of four distinct models of AF-induced electrical remodelling enabled us to carry out the first comprehensive study of electrophysiological consequences of remodelling at the entire 3D atria level (Figs 6–10). Previous models accounting for AF-induced remodelling of the 3D human atria (Kharche & Zhang, 2008) have not incorporated AP heterogeneity. The present model integrates the most complete set of regional cell models (Fig. 2) with AF-induced remodelling models (Fig. 3), allowing for the investigation of the pro-arrhythmic effects of regional AP heterogeneity in the electrically remodelled atria (Figs 6–10).

### Role of remodelled ionic currents

In our simulations, four different models of AF-induced electrical remodelling of the atria (AF-1 – AF -4) were considered to take into account various changes to seven major ion channel currents (see Table 1) as identified in various experimental studies. The simulation results from the four AF models showed qualitatively similar effects of the AF-induced electrical remodelling on APD abbreviation, APD dispersion and AF susceptibility. However, in quantitative terms each model showed different effects of the remodelling on tissue vulnerability to conduction block and upon the long-term dynamic behaviour of re-entry. These quantitative differences among the AF-1 to AF-4 variant AF models can be attributed to the differing degrees of remodelling of ionic currents that were considered in each model; this may reflect the relative contributions of the individual roles of different remodelled ionic currents in AF genesis and maintenance.

All four AF models considered up-regulation in *I*_K1_ and a down-regulation in *I*_CaL_, which were found to play an important role in APD abbreviation, resulting in a shorter wavelength of excitation waves that facilitated the initiation and maintenance of re-entry. This observation is consistent with a previous study (Zhang *et al.* 2005) in identifying the remodelled *I*_K1_ and *I*_CaL_ as primary contributors to atrial APD abbreviation. Our simulations also showed that without considering *I*_Kur_ remodelling (e.g. in AF-1 and AF-2 cases), the APD abbreviation was more pronounced as compared to the case when *I*_Kur_ remodelling was considered (e.g. in AF-3 and AF-4 cases). However, when *I*_Kur_ remodelling was incorporated (in AF-3 and AF-4 cases), the computed regional APD heterogeneity at CT/PM and PV/LA regions was larger, resulting in a greater tissue vulnerability to the initiation of re-entry (Fig. 7). In the latter cases, re-entrant excitation waves also broke down leading to the formation of multiple re-entrant wavelets. These findings suggest an important role of remodelled *I*_Kur_ in promoting AF, which is consistent with experimental findings (Burashnikov & Antzelevitch, 2008).

### Effects of atrial tissue segmentation

To improve the 3D structural representation of the atria, further segmentation of the anatomical model based on the Visible Human Dataset was added in agreement with recent patient-specific human models (Dössel *et al.* 2012): in particular, the segmentation of the PVs to incorporate the electrophysiologically distinctive PV model in the 3D atrial model, which was essential for investigation of the effect of PV/LA heterogeneity on the development of re-entrant excitation patterns linked with AF (Figs 8–10).

The segmentation of the anatomical model was performed manually due to the lack of detailed histological data associated with the Visible Human Female dataset. Nevertheless, this segmentation was guided by previous 3D anatomical models of the human, canine and sheep atria (Dössel *et al.* 2012; Aslanidi *et al.* 2013*b*; Zhao *et al.* 2013), and agrees well with those models. To investigate possible effects of varying the relative extent of segmented regions on simulation results, test simulations were also run using a variant model that considered less extensive PV sleeves in the PV region as compared to the original model (see Supplemental Fig. S14). Results from this variant model showed no change of the VW_CB_ and only a small change of the VW_R_ (<3 ms) as compared to the original segmentation. In addition, a variant model with an alteration to the defined CT region had no measurable effect on the measured VW_R_ either.

We assumed a stepwise change in electrical properties between two distinctive regions. However, it is possible that cells at the junction of two distinctive regions have graded changes in cellular electrophysiology or an interweaving distribution of different cell types. In our simulations, the use of a sequence of pre-conditioning stimuli (10 beats at a cycle length of 350 ms, see Methods) ensured that the resultant functional APD dispersion between two neighbouring regions showed a smooth spatial gradient, rather than a stepwise change, with the smoothing effects arising from the electrotonic interaction between cells. Such a smoothing effect has been demonstrated previously for transmural APD differences (Benson *et al.* 2008). To investigate possible effects of a graded change in cellular electrophysiology at the junction, simulations were also performed by using a 3D atrial wedge model in two conditions: one with and the other without considerations of gradient changes in cellular electrophysiological properties (for details see Online Supplement; eqn S31) between two distinctive tissue regions. No significant differences in the measured APD dispersion and tissue VWs were observed in the two cases (see Supplemental Fig. S15).

### Limitations

Electrophysiological data on which regional cell models are based are limited, particularly for the kinetics of ionic currents in the human atria. For this reason, canine data were used for situations in which the respective human data were missing. The absence of particular experimental data sets is a common limitation of modelling studies accounting for regional heterogeneity in the human atria, and, in common with the present investigation, the approach of incorporating data from alternative species has been implemented in previous studies (Courtemanche *et al.* 1998; Seemann *et al.* 2006; Aslanidi *et al.* 2011*b*; Dorn *et al.* 2012). It is encouraging that such an approach has resulted in models that can replicate regional APD and AP morphology differences observed in both the human and the canine atria (Feng *et al.* 1998; Burashnikov *et al.* 2004; Katoh *et al.* 2005; Gong *et al.* 2008). It should be noted that major results of the present study were confirmed by replacing the updated CRN-based models with a different family of human atrial AP models developed recently by Dorn *et al.* (2012). This suggests that our results and conclusions are not model dependent.

Recent modelling studies have provided evidence that accumulation of intracellular ions and *I*_NaK_ may play a significant role in modulating atrial rate-dependent repolarisation (Aslanidi *et al.* 2009; Grandi *et al.* 2011). These factors were not considered in the present study, but the possible pro-arrhythmic effects of accumulation of intracellular ions warrants further investigation in the future.

In our simulations, we also assumed a homogeneous AF-induced electrical remodelling on all regions within the atria. Recent studies have shown small differences in the effects of AF-induced electrical remodelling between the RA and LA (Caballero *et al.* 2010). The limited available data in this regard were not incorporated into the current model, but do need to be accounted for when further experimental data become available. Similarly, the present study considered only a uniform decrease in the diffusion coefficient that mimics AF-induced connexin remodelling in the atria. This consideration has certain limitations as AF-induced connexin remodelling is heterogeneous in the atrial tissue (van der Velden and Jongsma, 2002). It has been shown that such an inhomogeneous connexin remodelling can contribute towards wave breakdown (Zhang *et al.* 2009). However, the aim of the current study was to investigate the role of regional electrical heterogeneity in the development and behaviour of re-entry during AF in electrically normal and remodelled conditions. Therefore, an anisotropic and heterogeneous distribution of connexin expression was omitted in the model to facilitate interpretation of the data obtained: under the conditions of this study any observed wave breakdown would be attributable only to regional APD/ERP differences.

The model also contains only idealised fibres along the definitive bundles of the CT, PM and BB. As a result, the presence of fibre anisotropy plays little role in the long-term behaviour of AF in the model, and does not contribute to wave breakdown: in both isotropic and anisotropic conditions, a similar degree of wave breakdown is observed for each AF model. However, conduction patterns are altered by fibre anisotropy, with anisotropic conditions resulting in more irregular re-entrant patterns. Incorporation of detailed fibre structure throughout the atria would be anticipated to enable further investigations of the relative roles of electrical and structural factors in AF. The latter include anisotropic fibre arrangement (Aslanidi *et al.* 2013*a*), fibrosis and heterogeneous atrial wall thickness (Yamazaki *et al.* 2012).

### Conclusions

In this study we have investigated the role of regional heterogeneity in the generation and progression of re-entry in control and remodelled atria. It was shown that AF-induced electrical remodelling increases atrial susceptibility to arrhythmia due to increased tissue vulnerability and shortened APD, which respectively facilitate initiation and maintenance of re-entrant excitation waves.

## Abbreviations

1D, 2D, 3D: one-, two- and three-dimensional model
AF: atrial fibrillation
AF-0: atrial fibrillation model in which intercellular coupling is reduced but no changes to the electrical action potential are included
AF-1 – AF-4: atrial fibrillation-induced ion channel remodelling models
AP: action potential
APD: action potential duration
APD_90_: APD values at 90% repolarisation
APDr: APD restitution curve
AS: atrial septum
AVR: atrio-ventricular ring
BB: Bachmann's bundle
BCL: basic cycle length
*C*_m_: membrane capacitance
CRN model: Courtemanche–Ramirez–Nattel model of electrical action potential of human atrial cells
CT: crista terminalis
*D*: diffusion coefficient characterising the intercellular electrical coupling via gap junctions
ERP: effective refractory period
*F*_fbR_: factor characterising vulnerability to re-entry
*I*_CaL_: L-type calcium channel current
*I*_ion_: total ionic current flowing across the cell membrane
*I*_K1_: inward rectifier potassium channel current
*I*_Ks_: slow delayed rectifier potassium channel current
*I*_Kr_: rapid delayed rectifier potassium channel current
*I*_Kur_: ultra-rapid delayed rectifier potassium channel current
*I*_Na_: sodium channel current
*I*_NaCa_: sodium–calcium exchanger current
*I*_to_: transient outward potassium channel current
KM model: Koivumäki *et al.* model of electrical action potential of human atrial cells
LA: left atrium
LAA: left atrial appendage
PM: pectinate muscle
PV: pulmonary vein
RA: right atrium
RAA: right atrial appendage
SAN: sino-atrial node
SR: sinus rhythm
*V*_m_: cell membrane potential
VW_CB_: vulnerable window (time interval) for unidirectional conduction block
VW_R_: vulnerable window (time interval) for re-entry
