# Supplementary material for: Pro-arrhythmogenic effects of atrial fibrillation-induced electrical remodelling: insights from the three-dimensional virtual human atria
Source: J Physiol. 2013 Jun 3;591(Pt 17):4249–72. doi: 10.1113/jphysiol.2013.254987 (PMC3779115; doi:10.1113/jphysiol.2013.254987)
Supplement: Supplementary file 1 [file tjp0591-4249-SD1.zip › Colman_et_al_Online_Supplement_R1_Final.doc]

**Pro-arrhythmogenic Effects of Atrial Fibrillation Induced Electrical Remodelling–**

**Insights from 3D Virtual Human Atria**

Michael A. Colman, Oleg V. Aslanidi, Sanjay Kharche, Mark R. Boyett,

Clifford Garratt, Jules C. Hancox, Henggui Zhang

# Online Supplement

## 1. Ionic model of atrial cells

We incorporate recently developed formulations of I_Kur_ and I_to_[1] into the Courtemanche-Ramirez-Nattel (CRN) model [2] of the human atrial action potential (AP). The magnitude of I_CaL_ is also increased, to counteract the larger repolarising effect of the new formulation of I_Kur_ and to result in validated Ca^2+^ transient magnitudes. The magnitude is increased according to data which describes the magnitude of I_CaL_ in the human right atrial appendage to be between ~3 pA/pF and ~9.7 pA/pF [3–6]. A current density of ~8.5 pA/pF was chosen. Formulations of I_to_ and I_Kur_ are identical to those presented in Maleckar*et al.*[1].

## 2. Intracellular model structure and behaviour

We incorporate the intracellular calcium system developed by Koivumaki*et al.*[7](‘KM’ model) into the modified CRN cell model. We modify this system to make it more suitable for large scale simulations; the multi compartment model is reduced to just two compartments, representing the peripheral and interior sites of the cell. It was decided that this level of complexity is sufficient to model the behaviour required for this study, as it replicated such behaviour as larger Ca^2+^ transients in the periphery compared to the centre (Figure S1) and preserved Ca^2+^ transient behaviour (Ca^2+^ transient shape, the two-phase upstroke and high dependence on I_rel_).

Further details follow:

The intracellular space is divided into the junctional, or subspace (SS), compartment, into which the sarcolemmal currents act and on the Ca^2+^concentration of which the L-type calcium channel depends, and the non-junctional (NJ) intracellular space, as has been done previously in ventricular myocyte models [8,9].

The SS occupies the same proportional volume of the total cell as that of the KM model, and hence is scaled to fit the differences in cell size between the CRN and KM models. The volume of the intracellular space, NJ, is increased to occupy that of the total volume of the four intracellular spaces modelled in the KM model, as well as also being adjusted to the difference in cell size. The SS and NJ volumes are therefore 99.84 μm^3^ and 13,568.16 μm^3^, respectively. These add to give the total intracellular volume of 13,668 μm^3^, as in the original CRN model [2]. The SR space is divided into two compartments, each one interacting with either the SS (SR_SS_) or NJ (SR_NJ_) spaces via RyR, SR leak and SERCA processes.

The volumes of the SR compartments are adjusted as with the intracellular spaces: the volume of SR_SS_ is adjusted for cell size and the volume of SR_NJ_ is adjusted to occupy the full volume of the other three SR spaces used in KM, and then adjusted for cell size. The volumes of the SR_SS_ and SR_NJ_ spaces are therefore 301 μm^3^ and 903 μm^3^, respectively, giving a total SR volume of 1,204 μm^3^, in accordance with the total size of the SR in the CRN model (given as the addition of the NSR and JSR spaces)[2]. Ca^2+^ diffusion is allowed between the two intracellular spaces and the two SR spaces, and is modelled in the same way as in the KM model [7].

The system of equations describing the intracellular system are therefore as follows:

for x = SS, NJ:

#### I. SR uptake and release units

V_SS_	= 99.84 μm^3^

V_NJ_	= 13,568.16 μm^3^

SERCA

k_1_	= 7.5 x 10^6^

k_2_	= 0.468750

k_3_	= 2.314815

k_4_	= 7.5

C_pumps_	= 4.0 X 10^-2^

*Bulk -> SERCA*

$$J_{SERCA\_BULK,x}=V_{x}\left\{\left(k_{1}\left[Ca^{2+}\right]_{i,x}^{2}\left(c_{pumps}-\left[Ca^{2+}\right]_{SERCA,x}\right)\right.\right.$$

$$\left.-k_{2}\left[Ca^{2+}\right]_{SERCA,x}\right\}$$

*SERCA -> SR*

$$J_{SERCA\_SR,x}=V_{x}\left\{-k_{3}\left[Ca^{2+}\right]_{SR,x}^{2}\right.\left(c_{pumps}-\left[Ca^{2+}\right]_{SERCA,x}\right)$$

$$\left.+k_{4}\left[Ca^{2+}\right]_{SERCA,x}\right\}$$

$$\frac{d\left[Ca^{2+}\right]_{SERCA,x}}{dt}=\frac{J_{SERCA\_BULK,x}-J_{SERCA\_SR,x}}{V_{x}}$$

RyR

τ_RyRadapt_	= 1

τ_RyRact_	= 5 X 10^-3^

τ_RyRinact_	= 15 X 10^-3^

ν_SS_	= 625

ν_NJ_	= 1

$$J_{rel,x}=\upsilon_{x}V_{x}RyR_{o,x}RyR_{c,x}RyR_{SRCa,x}\left(\left[Ca^{2+}\right]_{SR,x}-\left[Ca^{2+}\right]_{i,x}\right)$$

$$RyR_{SRCa,x}=1-\left(1+\exp\left\{\left(\left[Ca^{2+}\right]_{SR,x}-0.3\right)/0.1\right\}\right)^{-1}$$

$$RyR_{a\infty,x}=0.505-0.427\left(1+\exp\left\{\left(1000\left[Ca^{2+}\right]_{i,x}-0.29\right)/0.082\right\}\right)^{-1}$$

$$RyR_{o\infty,x}=1-\left(1+\exp\left\{\left(1000\left[Ca^{2+}\right]_{i,x}-\left(RyR_{a.x}+0.22\right)\right)/0.03\right\}\right)^{-1}$$

$$RyR_{c\infty,x}=\left(1+\exp\left\{\left(1000\left[Ca^{2+}\right]_{i,x}-\left(RyR_{a.x}+0.02\right)\right)/0.01\right\}\right)^{-1}$$

$$\frac{dRyR_{a,x}}{dt}=\frac{RyR_{a\infty,x}-RyR_{a.x}}{\tau_{RyRadapt}}$$

$$\frac{dRyR_{o,x}}{dt}=\frac{RyR_{o\infty,x}-RyR_{o,x}}{\tau_{RyRact}}$$

$$\frac{dRyR_{c,x}}{dt}=\frac{RyR_{c\infty,x}-RyR_{c.x}}{\tau_{RyRinact}}$$

SR Ca^2+^ leak

k_SRleak_	= 6 X 10^-3^

$$J_{SRleak,x}=k_{SRleak}\left(\left[Ca^{2+}\right]_{SR,x}-\left[Ca^{2+}\right]_{i,x}\right)V_{x}$$

#### II. Intracellular and SR Ca^2+^ dynamics

A_SS-NJ_	= 2,492.324412 μm^2^

X_SS-NJ_	= 3.26 μm

D_Ca_	= 780 μm^2^s^-1^

SL_low_	= 165

SL_high_	= 13

B_Ca_	= 2.4 X 10^-2^

[CSQN]	= 6.7

K_dSLlow_	= 1.1

K_dSLhigh_	= 1.3 X 10^-2^

K_dBCa_	=2.38 X 10^-3^

K_dCSQN_	= 0.8

Ca^2+^ diffusion between SS and NJ

$$J_{SS-NJ}=D_{Ca}\frac{A_{SS-NJ}}{X_{SS-NJ}}\left(\left[Ca^{2+}\right]_{SS}-\left[Ca^{2+}\right]_{NJ}\right)$$

Intracellular Ca^2+^ fluxes

$$J_{Ca,SS}=-J_{SS-NJ}+J_{SRleak,SS}-J_{SERCA\_BULK,SS}+J_{rel,SS}$$

$$J_{Ca,NJ}=J_{SS-NJ}+J_{SRleak,NJ}-J_{SERCA\_BULK,NJ}+J_{rel,NJ}$$

SR Ca^2+^ fluxes

$$J_{SRCa,x}=J_{SERCA\_SR,x}-J_{SRleak,x}-J_{rel,x}$$

Ca^2+^ buffers

$$\beta_{SS}=\left\{1+\frac{SL_{low}K_{dSLlow}}{\left(\left[Ca^{2+}\right]_{i,ss}+K_{dSLlow}\right)^{2}}+\frac{SL_{high}+K_{dSLhigh}}{\left(\left[Ca^{2+}\right]_{i,ss}+K_{dSLhigh}\right)^{2}}\right.$$

$$+\left.\frac{B_{Ca}K_{dBCa}}{\left(\left[Ca^{2+}\right]_{i,ss}+K_{dBCa}\right)^{2}}\right\}^{-1}$$

$$\beta_{i,NJ}=\left\{1+\frac{B_{Ca}K_{dBCa}}{\left(\left[Ca^{2+}\right]_{i,NJ}+K_{dBCa}\right)^{2}}\right\}^{-1}$$

$$\beta_{SR,x}=\left\{1+\frac{[CSQN]K_{dCSQN}}{\left(\left[Ca^{2+}\right]_{SR,NJ}+K_{dCSQN}\right)^{2}}\right\}^{-1}$$

[Ca^2+^] differential equations

$$\frac{d[Ca^{2+}]_{i,SS}}{dt}=\beta_{SS}\left(\frac{J_{Ca,SS}}{V_{SS}}+\frac{-I_{CaL}-I_{Ca,b}-I_{Ca,p}+2I_{NaCA}}{2V_{ss}F}\right)$$

$$\frac{d\left[Ca^{2+}\right]_{i,NJ}}{dt}=\frac{\beta_{i,NJ}J_{Ca,NJ}}{V_{NJ}}$$

$$\frac{d\left[Ca^{2+}\right]_{SR,NJ}}{dt}=\beta_{SR,NJ}D_{CaSR}\left(\frac{\left[Ca^{2+}\right]_{SR,SS}-\left[Ca^{2+}\right]_{SR,NJ}}{\Delta r^{2}}\right.$$

$$+\left.\frac{\left[Ca^{2+}\right]_{SR,NJ}-\left[Ca^{2+}\right]_{SR,SS}}{6\Delta r^{2}}\right)+\frac{\beta_{SR,NJ}J_{SRCa,NJ}}{V_{SR,NJ}}$$

$$\frac{d\left[Ca^{2+}\right]_{SR,SS}}{dt}=\beta_{SR,SS}D_{CaSR}\left(\frac{\left[Ca^{2+}\right]_{SR,NJ}-\left[Ca^{2+}\right]_{SR,SS}}{\Delta r^{2}}\right.$$

$$+\left.\frac{\left[Ca^{2+}\right]_{SR,SS}-\left[Ca^{2+}\right]_{SR,NJ}}{8\Delta r^{2}}\right)+\frac{\beta_{SR,SS}J_{SRCa,SS}}{V_{SR,SS}}$$

Where, in all of the above, [Ca^2+^]_i,x_ is the intracellular Ca^2+^ concentration in compartment x, [Ca^2+^]_SR,x_ is the SR Ca^2+^ concentration in compartment SR_x_, [Ca^2+^]_SERCA,x_ is the Ca^2+^ concentration held in SERCA between the intracellular compartment x and SR compartment SR_x_, and V_x_ is the volume of the intracellular compartment x.

Note that any current formulations or reversal potential in the CRN model which depend on [Ca^2+^]_i_, this term must now be replaced by [Ca^2+^]_ss_.

**Figure S1 – Intracellular structure of the CRN model variant.**

A. Intracellular structure, B. Mean Ca^2+^ transient and C. Compartmental Ca^2+^ transients for the KM model (i) and updated CRN model (ii). LTCC in (A) stands for L-type Calcium Channels. SS is subspace and NJ is non-junctional compartments. Panels Bi and Ci are modified from [7]

## 3. Comparison of new CRN variant with original CRN model

The new model variant of the CRN model is similar to the original CRN model but with some marked differences. Firstly, the new model has a shorter APD and a less pronounced dome than the CRN model (Figure S2A). Secondly, the time course of phase 1 repolarisation is slower in the new variant than the original model and the notch potential is slightly more negative (Figure S2A-insert). These differences can be attributed to the introduction of the new formulations of I_Kur_ and I_to_ respectively (Figure S2B,D). The new model has an increased dependence on I_Kur_ and I_CaL_ compared to the original CRN model (Figure S2C,D). The Ca^2+^ transient is also now similar to the KM model (Figure S1), which is markedly different to that of the CRN model. The model also exhibits improved ionic stability compared to the CRN model (Figure S2E).

**Figure S2 – Comparison of AP and current properties CRN variant with CRN model**

A. AP traces from the CRN model (grey) and the updated CRN model (black). The inset is zoomed on phase 1 repolarisation. B-D Current traces for I_to_ (B), I_CaL_ (C) and I_Kur_ (D) during an AP. E. APD_90_ drift over 10 minutes between the two models. Each is normalised to its APD_90_ at the start of the pacing train.

**Figure S3 – validation of new model**

A. AP produced by the updated CRN model, compared to experimental traces (B) from [1,3,4,10–13]. C. AP traces focussed on phase 1 repolarisation between the model (black line) and experimental traces from [3,4,10,11,13]. D. APD restitution of the model compared with experimental data from [12,14,15].

## 4. Validation of new CRN variant

The new variant of the CRN cell model matches well with available experimental data in terms of morphology (Figure S3A-C) and restitution properties (Figure S3D), and the APD falls within experimentally observed ranges (Table S1). The primary improvement on the CRN model, other than Ca^2+^ dynamics, is in the agreement of phase 1 repolarisation: despite a large range of morphological differences in the available experimental traces, many have very similar time courses of phase 1 repolarisation, to which the model matches well (Figure S3C).

**Table S1 – Validation of APD_90_ of the** new model

| **Study** | **APD_90_ (ms)(1 Hz)** |
| --- | --- |
| **Model** | 242 |
| **Pau *et al.* 2007 [11]** | 239 ± 11 |
| **Redpath *et al.* 2006 [4]** | 210 ± 17 |
| **Katoh *et al.* 2005[16]** | 255 ± 39 |
| **Dawodu *et al.* 1996 [15]** | 361 ± 71 |
| **Bosch *et al.* 1999 [12]** | 255 ± 45 |
| **Dobrev *et al.* 2002 [14]** | 203 ± 10 |
| **Kim *et al.* 2002 [17]** | 258 ± 25 |

## 5. Development of regional cell models

Development of regional cell models is based on a range of different datasets and described for each region individually below. A summary of all of the modifications for each cell model are shown in table S2.

**Pectinate muscle (PM):** As with the original CRN model, the CRN model variant shares morphology with the PM. However, the magnitude of I­_CaL_ is based on data from the human right atrial appendage (RAA). It is shown in canine that the PM has a magnitude of I_CaL_ of 1/1.06 times that of the RAA [18], and hence the PM model is modified accordingly. Such a modification has a small effect on the AP and still resembles that of the experimental PM trace (Figure S4).

**Right atrial appendage (RAA):** A model for the RAA is developed based on electrophysiological data for human from Gong *et al*. 2008[19]. The magnitude of I_to_ in the RAA is significantly smaller than that of the CRN model variant. A good fit to the I-V curve is achieved through a 60% reduction in the maximum conductance of I_to_ and a small modification to the gradient of the steady state of activation (Figure S5A). This is consistent with the data from Feng*et al.* 1998 [18] in canine, in which I_to_ in the RAA is observed to have a magnitude of 50% that of the PM with similar kinetics. The I-V relationship for I_Kur_in experiment displays both a smaller magnitude and different kinetics to that of

**Figure S4 – Pectinate muscle model**

A. AP trace from the updated CRN model (Baseline, black line) and PM model (dotted line) compared to an experimental trace from the PM [18] (B).

**Figure S5 – RAA I-V relationships**

I-V relationship for I_to_ (A) and I_Kur_ (B) in the updated CRN model (Baseline, dotted line) and RAA model (solid line) compared with experimental data [19] (points).

the CRN model variant (Figure S5B). It is necessary to modify the steady state formulations of both the activation and inactivation gates to fit these data. No modification to the maximum conductance is necessary to achieve a good fit between model and data. The formulations for I_to_ and I_Kur_ are as follows:

I_to_

g_to_ = 0.0784 nS/pF

$$r_{\infty}=\left[1+\exp\left(\left[V_{m}-1\right]/-13.2\right)\right]^{-1}$$

I_Kur_

$$a_{\infty}=\left[1+\exp\left(\left[V_{m}-8\right]/-18.3326\right)\right]^{-1}$$

$$i_{\infty}=\left[1+\exp\left(\left[V_{m}+32.5\right]/17.69\right)\right]^{-1}$$

Where all other equations are as they appear in the Maleckar *et al*. model [1]

**Crista terminalis (CT) and Atrio-ventricular ring (AVR):** In the absence of new electrophysiological data for human for either the CT or the AVR, models for these regions are incorporated exactly as they were in Seemann *et al.* 2006 [20] for the CRN model. This is based on electrophysiological data taken from canine [18]. Hence, for the CT, the magnitudes of I_CaL_ and I_to_ are increased by factors of 1.67 and 1.28, respectively. In the AVR, the magnitude of I_CaL_ is reduced by a factor of 0.67 and I_Kr_ is increased by a factor of 1.63. No kinetic changes are modelled.

**Atrial Septum (AS):** A model for the AS is developed based on electrophysiological data from Gong *et al* 2008 [19] for human. No significant difference in the I-V relationship for I_to_ between the AS and the RAA is observed. The formulation for I_to_ developed in the previous section for the RAA fits equally well with the data from the AS as it does the RAA (Figure S6A). A reduction in the maximum conductance of I_Kur_ relative to the RAA is also observed, with no significant kinetic changes (Figure S6B)[19]. Scaling the maximum conductance of the formulation of I_Kur_ developed for the RAA by a factor of 0.677 is sufficient to fit the I-V relationship (Figure S6B). I_CaL_ in the AS is only a quarter the magnitude of that observed in the RAA, and hence it is modelled as such. The magnitude of I_Na_ in the AS is observed to be 1.3 times greater than that of the RAA, and the time constants of both activation and inactivation are approximately double those in the RAA. Hence, these parameters are increased accordingly. It is also observed that the I-V relationships of I_K1_ for the RAA and the AS diverge at voltages positive of -80 mV, and converge at voltages positive of -30 mV. This results in a difference in the magnitude of I_K1_ at a voltage corresponding to the resting potential of 0.16 pA/pF and of 0.09 pA/pF at -30 mV. It is found that a shift in the voltage dependence of I_K1_ in the AS of -6 mV relative to the RAA closely replicates this behaviour. A difference in magnitude of 0.17 pA/pF results at a potential corresponding to the resting potential of the RAA and of 0.07 pA/pF at -30 mV. Hence, the formulations for I_to_, I_Kur_ and I_CaL_ in the AS model are identical to those in the RAA model,

**Figure S6 – AS I-V relationships**

I-V relationships for I_to_ (A) and I_Kur_ (B) for the AS model (solid line), RAA model (dotted line), AS experimental data (closed squares) and RAA experimental data (open squares) [19]

with the maximum conductances of I_Kur_ and I_CaL_ scaled by factors of 0.677 and 0.25, respectively. The formulations for I_K1_ and I_Na_ in the AS models are as follows:

I_K1_

$$I_{K1}=g_{K1}\left(V_{m}-E_{K}-6\right)/\left(1+\exp\left[0.07\left(V_{m}+74\right)\right]\right)$$

I_Na_

g_Na_= 10.14 nS/pF

$$\tau_{ac}=2/\left(\alpha_{ac}+\beta_{ac}\right)$$

$$\tau_{inac}=2/\left(\alpha_{inac}+\beta_{inac}\right)$$

Where all other equations are identical to that in the CRN model.

**Left Atrium (LA):** There is electrophysiological data on I_Kr_, I_Ks_ and I_to_ in canine provided by Li *et al*. 2001 [21] and Ehrlich *et al.* 2003 [22]. Due to the electrophysiological data on the potassium currents being from canine, it is necessary to normalise the I-V relationships in order to make them applicable for fitting into human models. Hence, the experimental I-V curves for the LA are normalised to the maximum current observed in the RA in canine. Similarly, model I-V curves in the LA are normalised to the maximum current observed in the I-V relationship in the CRN variant model. By this method, it was found that for I_to_, a reduction in the maximum conductance of 47% was sufficient to fit the data provided by Ehrlich *et al.* 2003 [22] (Figure S7A). To fit the data for I_Ks_, also provided by Ehrlich *et al.* 2003 [22], a shift in the voltage dependence of the steady of activation and a reduction in the gradient combined with an increase in the maximum conductance by a factor

**Figure S7: I-V relationships for the LA**

I-V relationships for Ito and IKs for the LA model (solid line) compared to the baseline model (dotted line) and experimental data [22] (points)

of 1.8 was necessary (Figure S7B).Li *et al.* 2001 [38] observed that the maximum conductance of I_Kr_ in the LA is 1.6 times greater than that of the RA, with no significant kinetic changes. Hence, this is modelled appropriately. The formulation for I_Ks_ is therefore:

$$xs_{\infty}=\sqrt{\left(1/1+\exp\left[\left(V_{m}-6.47\right)/-6.35\right]\right)}$$

**Left Atrial Appendage (LAA):** A LAA model is developed based on electrophysiological data from Caballero *et al.* 2010 [23] for the human LAA, in relation to the RAA. They identify I_to_ as having no significant differences in magnitude or kinetics in relation to the RAA, and hence the RAA model for I_to_ is used. I_Kur_ is also shown to have no significant kinetic differences between the LAA and RAA, and a reduction in the maximum conductance of 22% is observed. I_Ks_ is expressed to a greater degree by a factor of 1.5 in the LAA relative to the RAA, with no significant kinetic changes in the voltage range of -40 mV to +40 mV. Hence, the RAA models for I_Kur_ and I_Ks_ are used in the LAA model, but scaled by 0.78 and 1.5, respectively. It is assumed that all other currents are identical to that of the LA.

**Bachmann’s bundle (BB):** There is a lack of detailed electrophysiological data for the BB in either human or canine. Hence, data from rabbit must be used, as has been done in a previous modelling study in the canine atria [24]. There is, however, an AP trace available for the BB from canine, with which the morphology of the produced AP can be validated and on which modifications to the results observed in rabbit can be based. The Aslanidi *et al.* 2011 model [25] for the rabbit BB is used as a basis for developing a BB model in human.In this study, the maximum conductances of I_CaL_ and I_to_ are increased by a factor of 2.75 and 1.17, respectively, in the BB relative to the RA, and the maximum conductance of I_K1_ is reduced by a factor of 0.6. In canine, it was observed that smaller modifications to I_CaL_ and I_K1_ were required to achieve an AP which fell within experimental ranges[24]. In the present model, I_CaL_ is increased by a factor of 2.32 and I_K1_ is reduced by a factor of 0.85. I_to_ is modified exactly as in rabbit, with an increase of 1.17.

**Pulmonary Vein (PV):** The PV model used is incorporated exactly as in Jones *et al.* 2012 [26], based on the CRN model. This model is fit to data available from canine, and involves a shift of the voltage dependence of the steady state of activation of I_Kr_ of -4.2 mV and I_Ks_ of -9.6 mV, as well as a decrease in the gradient of the steady state of activation of I_Kr_ by a factor of 0.938 and an increase of the gradient of I_Ks_ of 1.047. It also involves a shift in the voltage dependence of I_K1_ of -15 mV.

Table S2 – Model parameters for regional cell models, compared to the baseline model

| **Region** | **Current** | **Modification** |
| --- | --- | --- |
| **PM** | I_CaL_ | g_CaL_ X 0.94 |
| **RAA** | I_to_  I_Kur_ | gto X 0.4, eqn.  eqn. , |
| **CT** | I_CaL_  I_to_ | g_CaL_ X 1.67  g_to_ X 1.28 |
| **AVR** | I_CaL_  I_Kr_ | g_CaL_ X 0.67  g_Kr_ X 1.63 |
| **AS (compared to RAA model)** | I_Kur_  I_CaL_  I_Na_  I_K1_ | g_Kur_ X 0.667  g_CaL_ X 0.25  g_Na_ X 1.3, τ_ac_, τ_inac_ X 2 (eqns. )  eqn. |
| **LA** | I_to_  I_Ks_  I_Kr_ | g_to_ X 0.53  g_Ks_ X 1.8, eqn.  g_Kr_ X 1.6 |
| **LAA (compared to RAA model)** | I_Kur_  I_Ks_  I_Kr_ | g_Kur_ X 0.78  g_Ks_ X 1.5  g_Kr_ X 1.6 |
| **BB** | I_CaL_  I_K1_  I_to_ | g_CaL_ X 2.2  g_K1_ X 0.85  g_to_ X 1.17 |

## 5. Regional cell model morphological agreement

The regional cell models replicate proportional APD differences observed in the atria, as is discussed in the main manuscript. Models also match well morphologically with available data, as is shown in Figures S8 – S12. One notable difference is in the LA and LAA (Figure S12). No AP recordings were available from both the LA and the LAA from a single experimental study, and hence it is not straightforward to compare APD differences in these regions. Therefore, it was assumed that the difference between the LA and LAA is similar to that between the RA and RAA. Moreover, even when a large LA/LAA heterogeneity was introduced in the present model, it did not produce a notable effect on excitation wave breakdown.

**Figure S8 - CT and AVR morphology comparison**

Comparison of morphology of the CT (A) and AVR (B) models (i) with experiment (ii) [18,27].

**Figure S9 - RAA morphology comparison**

Comparison of the morphology of the RAA model (A) with experimental traces (B) [1,12,18,19]

**Figure S-10 AS morphology comparison**

Comparison of the AS morphology (relative to the RAA) in model (A) and experiment (B) [19].

**Figure S11 - BB morphology comparison**

Comparison of the BB morphology (relative to the CT)in model (A) and experiment (B) [27]

**Figure S12 - LA morphology comparison**

Comparison of the LA and LAA models (A) with experimental traces (B) [21,28].

**Figure S13 – APD restitution curves for the regional cell models**

## 6. Segmentation investigations

The effect of varied definition of the PVs on the VW_CB_ and VW_R_was investigated. Results are shown in Figure S14. It was shown that the VW_CB_ was unaffected, and the VW­_R_ showed only a small change.

We also investigated the effect of smooth gradients in cellular electrophysiology between regions as opposed to a step-wise change at the boundary (Figure S15). For this, we used an idealised wedge of tissue, onto which a gradient map (F_cell_) was applied. The electrophysiology of a particular node is determined from its F_cell_ map (varying between 0 and 1) in such a way:

$$P=P_{1}+F_{cell}\left(P_{2}-P_{1}\right)$$

Where P is any parameter which is different between two neighbouring regions, and P_1_ and P_2_ are the values of that parameter in region 1 and 2. Hence, an F_cell_ value of 0 determines electrophysiology of identical to region 1 and an F_cell_ value of 1 determines electrophysiology identical to region 2.

**Figure S14 –** Effect of PV segmentation definition on vulnerability

The vulnerability windows (B) corresponding to different segmentations of the PVs (A, blue regions). No difference is observed in the VW_CB_ but differences are observed in the VW_R_.

**Figure S15 – The effects of electrophysiological gradients on vulnerability windows**

**A. Examples of three different gradient functions used in determining electrophysiological properties (i-iii). B. The VW_CB_ for discrete junction and the three gradients illustrated in A for the CT/PM junction, AF-4. Note that the total length of the slab is 3.3 cm.**

## References

1.	Maleckar MM, Greenstein JL, Giles WR, Trayanova NA. K+ current changes account for the rate dependence of the action potential in the human atrial myocyte. Am. J. Physiol. Heart Circ. Physiol. 2009 Oct;297(4):H1398–1410.

2.	Courtemanche M, Ramirez RJ, Nattel S. Ionic mechanisms underlying human atrial action potential properties: insights from a mathematical model. Am. J. Physiol. 1998 Jul;275(1 Pt 2):H301–321.

3.	Van Wagoner DR, Pond AL, Lamorgese M, Rossie SS, McCarthy PM, Nerbonne JM. Atrial L-type Ca2+ currents and human atrial fibrillation. Circ. Res. 1999 Sep 3;85(5):428–36.

4.	Redpath CJ, Rankin AC, Kane KA, Workman AJ. Anti-adrenergic effects of endothelin on human atrial action potentials are potentially anti-arrhythmic. J. Mol. Cell. Cardiol. 2006 May;40(5):717–24.

5.	Tipparaju SM, Kumar R, Wang Y, Joyner RW, Wagner MB. Developmental differences in L-type calcium current of human atrial myocytes. Am. J. Physiol. Heart Circ. Physiol. 2004 May;286(5):H1963–1969.

6.	Tsai C-T, Chiang F-T, Tseng C-D, Hwang J-J, Kuo K-T, Wu C-K, et al. Increased expression of mineralocorticoid receptor in human atrial fibrillation and a cellular model of atrial fibrillation. J. Am. Coll. Cardiol. 2010 Feb 23;55(8):758–70.

7.	Koivumäki JT, Korhonen T, Tavi P. Impact of sarcoplasmic reticulum calcium release on calcium dynamics and action potential morphology in human atrial myocytes: a computational study. PLoS Comput. Biol. 2011;7(1):e1001067.

8.	O’Hara T, Virág L, Varró A, Rudy Y. Simulation of the undiseased human cardiac ventricular action potential: model formulation and experimental validation. PLoS Comput. Biol. 2011 May;7(5):e1002061.

9.	Decker KF, Heijman J, Silva JR, Hund TJ, Rudy Y. Properties and ionic mechanisms of action potential adaptation, restitution, and accommodation in canine epicardium. Am. J. Physiol. Heart Circ. Physiol. 2009 Apr;296(4):H1017–1026.

10.	Wettwer E, Hála O, Christ T, Heubach JF, Dobrev D, Knaut M, et al. Role of IKur in Controlling Action Potential Shape and Contractility in the Human Atrium Influence of Chronic Atrial Fibrillation. Circulation. 2004 Oct 19;110(16):2299–306.

11.	Pau D, Workman AJ, Kane KA, Rankin AC. Electrophysiological and arrhythmogenic effects of 5-hydroxytryptamine on human atrial cells are reduced in atrial fibrillation. J. Mol. Cell. Cardiol. 2007 Jan;42(1):54–62.

12.	Bosch RF, Zeng X, Grammer JB, Popovic K, Mewis C, Kühlkamp V. Ionic mechanisms of electrical remodeling in human atrial fibrillation. Cardiovasc. Res. 1999 Oct;44(1):121–31.

13.	Workman AJ, Pau D, Redpath CJ, Marshall GE, Russell JA, Norrie J, et al. Atrial cellular electrophysiological changes in patients with ventricular dysfunction may predispose to AF. Heart Rhythm. 2009 Apr;6(4):445–51.

14.	Dobrev D, Ravens U. Remodeling of cardiomyocyte ion channels in human atrial fibrillation. Basic Res. Cardiol. 2003 May;98(3):137–48.

15.	Dawodu AA, Monti F, Iwashiro K, Schiariti M, Chiavarelli R, Puddu PE. The shape of human atrial action potential accounts for different frequency-related changes in vitro. Int. J. Cardiol. 1996 Jun;54(3):237–49.

16.	Katoh H, Shinozaki T, Baba S, Satoh S, Kagaya Y, Watanabe J, et al. Monophasic action potential duration at the crista terminalis in patients with sinus node disease. Circ. J. 2005 Nov;69(11):1361–7.

17.	Kim B-S, Kim Y-H, Hwang G-S, Pak H-N, Lee SC, Shim WJ, et al. Action potential duration restitution kinetics in human atrial fibrillation. J. Am. Coll. Cardiol. 2002 Apr 17;39(8):1329–36.

18.	Feng J, Yue L, Wang Z, Nattel S. Ionic mechanisms of regional action potential heterogeneity in the canine right atrium. Circ. Res. 1998 Sep 7;83(5):541–51.

19.	Gong D, Zhang Y, Cai B, Meng Q, Jiang S, Li X, et al. Characterization and comparison of Na+, K+ and Ca2+ currents between myocytes from human atrial right appendage and atrial septum. Cell. Physiol. Biochem. 2008;21(5-6):385–94.

20.	Seemann G, Höper C, Sachse FB, Dössel O, Holden AV, Zhang H. Heterogeneous three-dimensional anatomical and electrophysiological model of human atria. Philos Transact A Math Phys Eng Sci. 2006 Jun 15;364(1843):1465–81.

21.	Li D, Zhang L, Kneller J, Nattel S. Potential ionic mechanism for repolarization differences between canine right and left atrium. Circ. Res. 2001 Jun 8;88(11):1168–75.

22.	Ehrlich JR, Cha T-J, Zhang L, Chartier D, Melnyk P, Hohnloser SH, et al. Cellular electrophysiology of canine pulmonary vein cardiomyocytes: action potential and ionic current properties. J. Physiol. (Lond.). 2003 Sep 15;551(Pt 3):801–13.

23.	Caballero R, De la Fuente MG, Gómez R, Barana A, Amorós I, Dolz-Gaitón P, et al. In humans, chronic atrial fibrillation decreases the transient outward current and ultrarapid component of the delayed rectifier current differentially on each atria and increases the slow component of the delayed rectifier current in both. J. Am. Coll. Cardiol. 2010 May 25;55(21):2346–54.

24.	Aslanidi OV, Butters TD, Ren CX, Ryecroft G, Zhang H. Electrophysiological models for the heterogeneous canine atria: computational platform for studying rapid atrial arrhythmias. Conf Proc IEEE Eng Med Biol Soc. 2011;2012:1693–6.

25.	Aslanidi OV, Robinson R, Cheverton D, Boyett MR, Zhang H. Electrophysiological substrate for a dominant reentrant source during atrial fibrillation. Conf Proc IEEE Eng Med Biol Soc. 2009;2009:2819–22.

26.	Jones G, Spencer B, Adeniran I, Zhang H. Development of Biophysically Detailed Electrophysiological Models of Pacemaking and Non-Pacemaking Human Pulmonary Vein Cardiomyocytes. Engineering in Medicine and Biology Society. 2012.

27.	Burashnikov A, Mannava S, Antzelevitch C. Transmembrane action potential heterogeneity in the canine isolated arterially perfused right atrium: effect of IKr and IKur/Ito block. Am. J. Physiol. Heart Circ. Physiol. 2004 Jun;286(6):H2393–2400.

28.	Yeh Y-H, Qi X, Shiroshita-Takeshita A, Liu J, Maguy A, Chartier D, et al. Atrial tachycardia induces remodelling of muscarinic receptors and their coupled potassium currents in canine left atrial and pulmonary vein cardiomyocytes. Br. J. Pharmacol. 2007 Dec;152(7):1021–32.
